# Supplementary figures and images for: Extra virgin olive oil mitigates lung injury in necrotizing enterocolitis: Effects on TGFβ1, Caspase-3, and MDA in a neonatal rat model
Source: PLoS One. 2025 Apr 15;20(4):e0320938. doi: 10.1371/journal.pone.0320938 (PMC11999148; doi:10.1371/journal.pone.0320938)

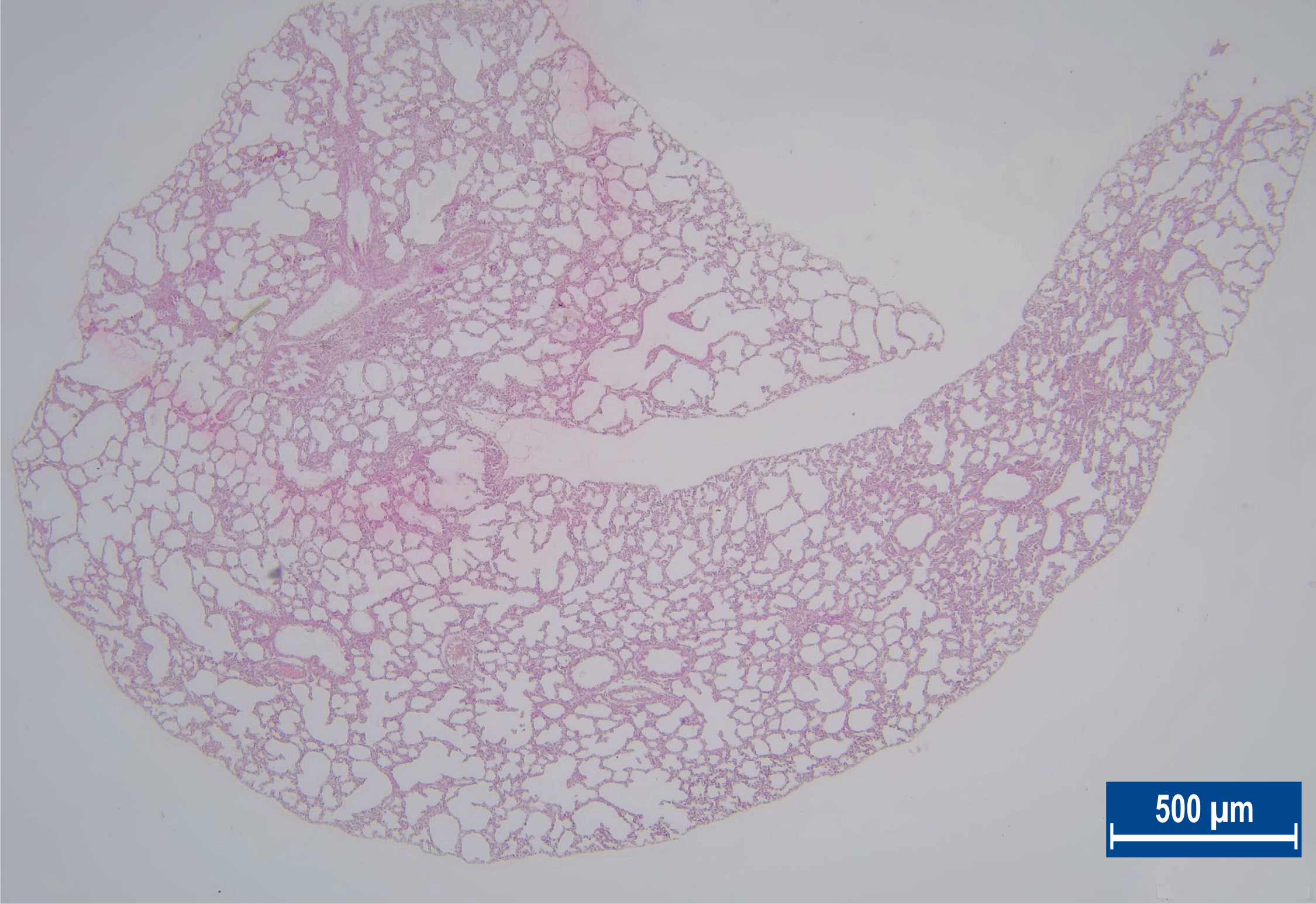

Supplement: S1 Fig — (TIF) [file pone.0320938.s001.tif]

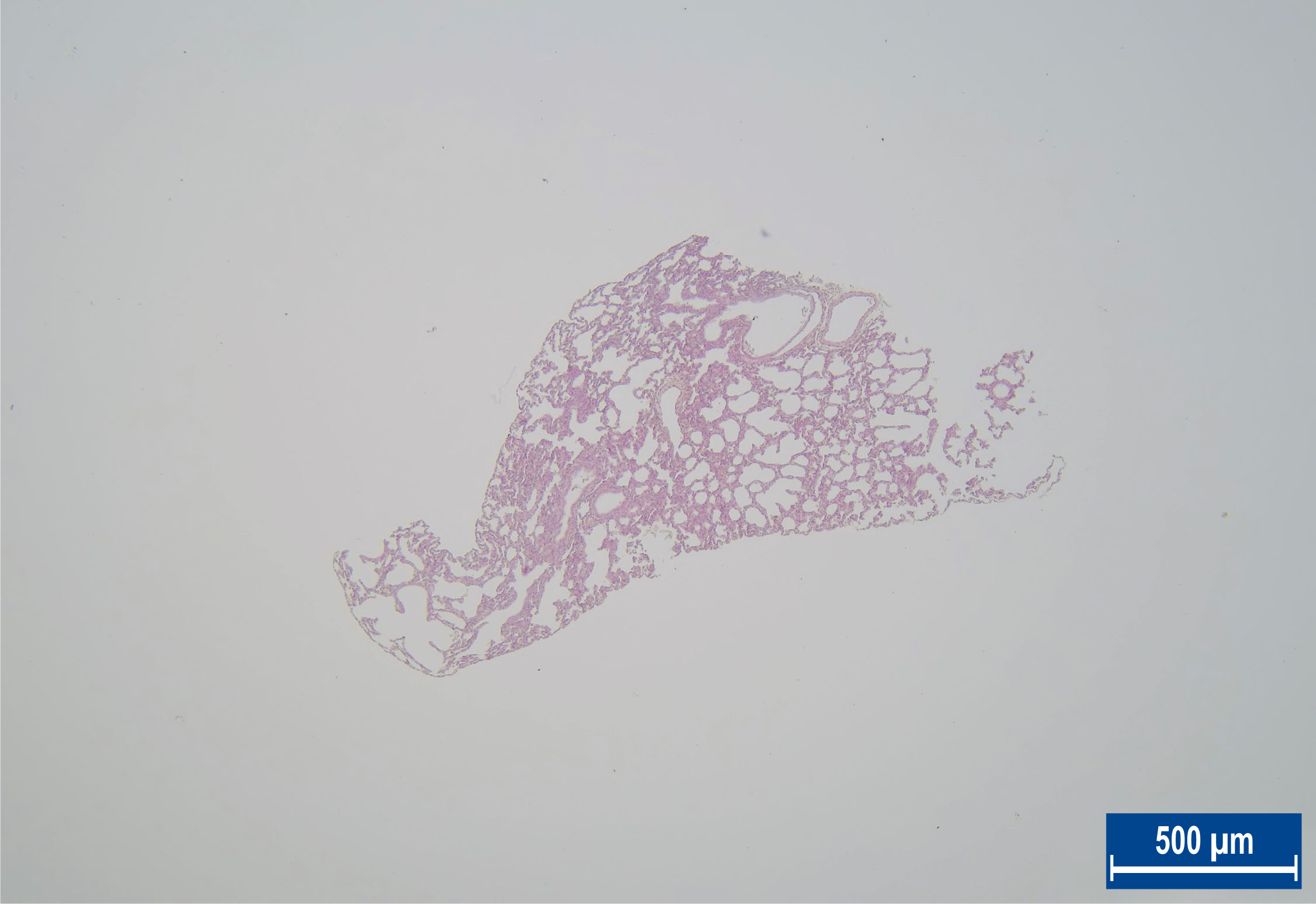

Supplement: S2 Fig — (TIF) [file pone.0320938.s002.tif]

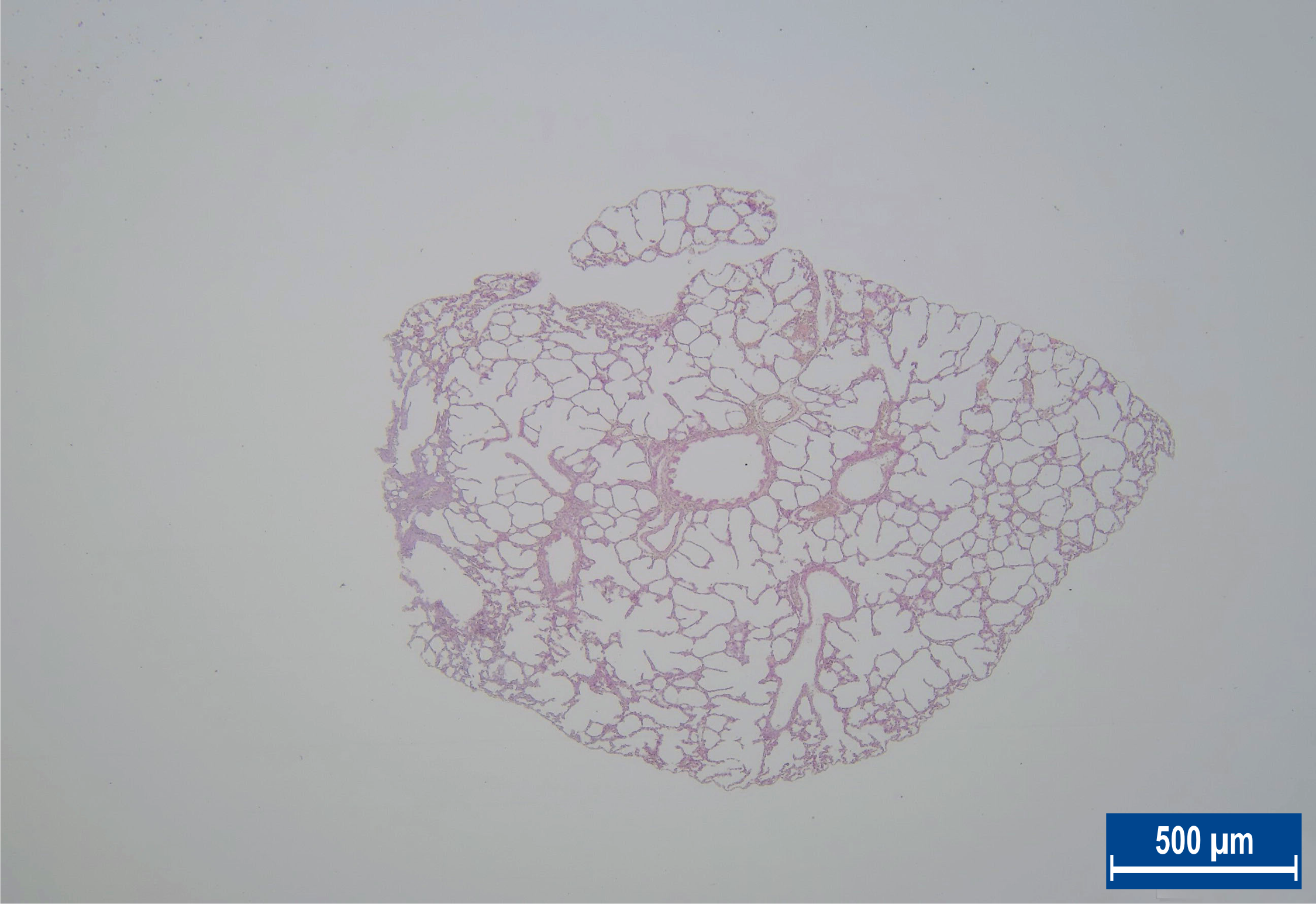

Supplement: S3 Fig — (TIF) [file pone.0320938.s003.tif]

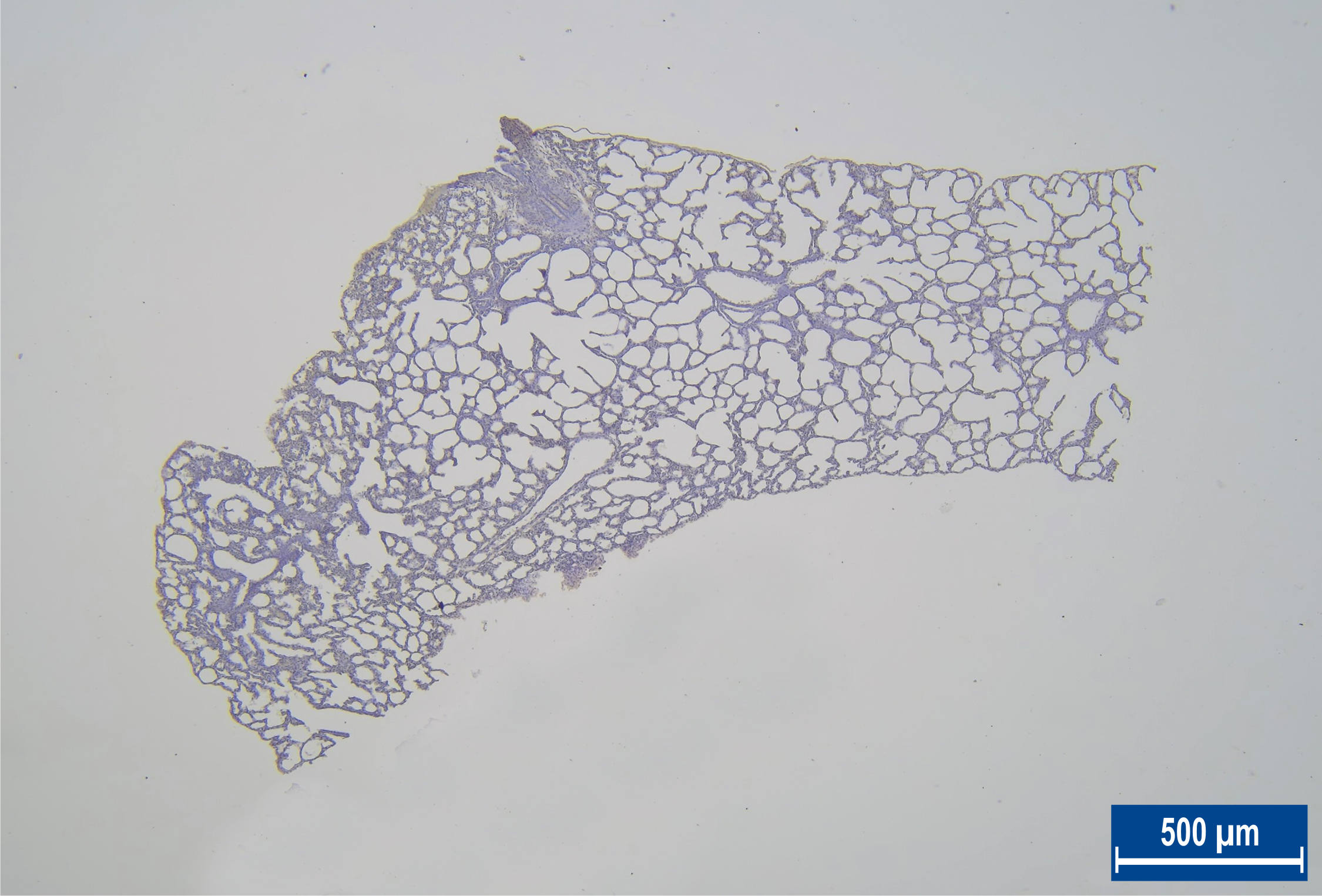

Supplement: S4 Fig — (TIF) [file pone.0320938.s004.tif]

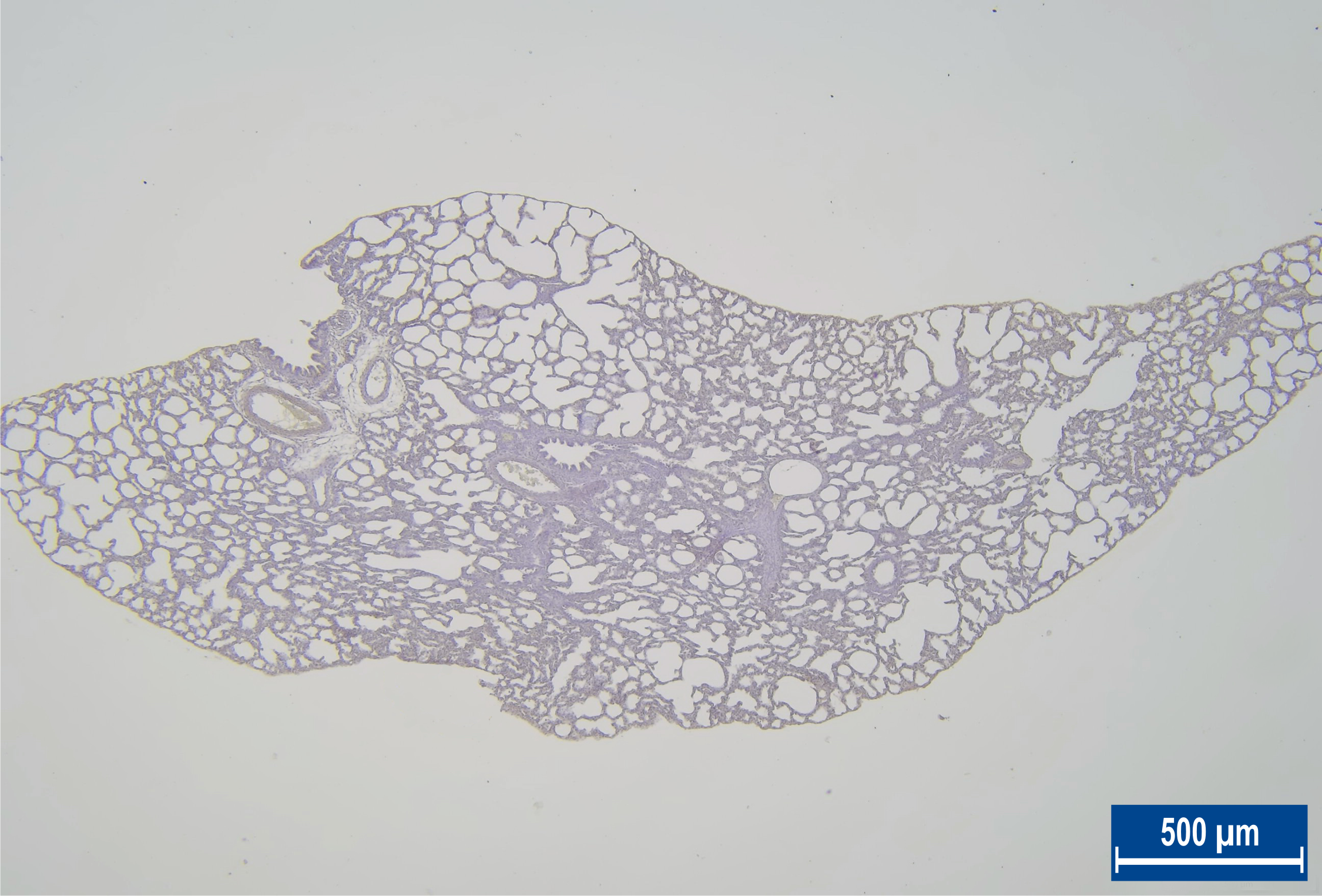

Supplement: S5 Fig — (TIF) [file pone.0320938.s005.tif]

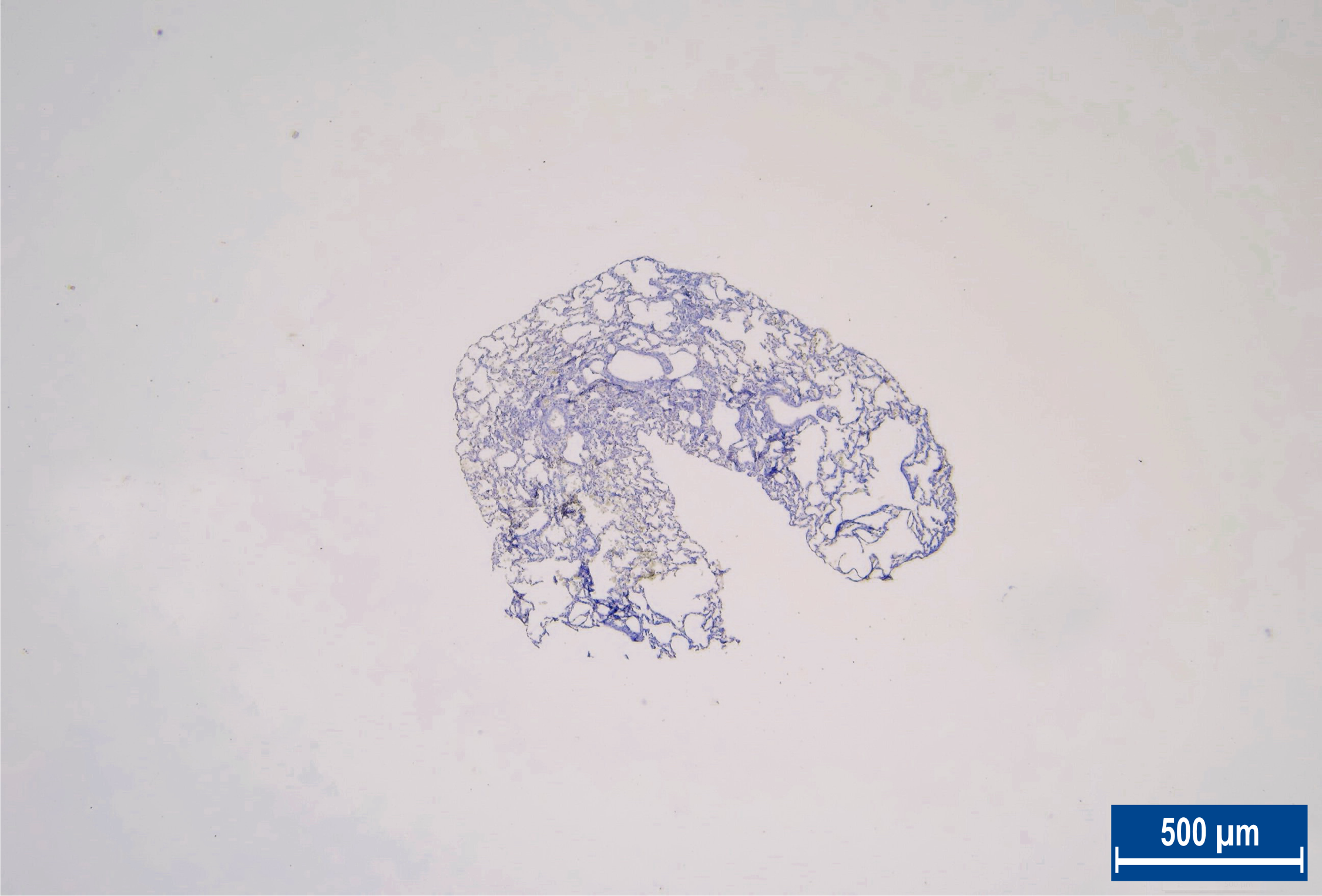

Supplement: S6 Fig — (TIF) [file pone.0320938.s006.tif]

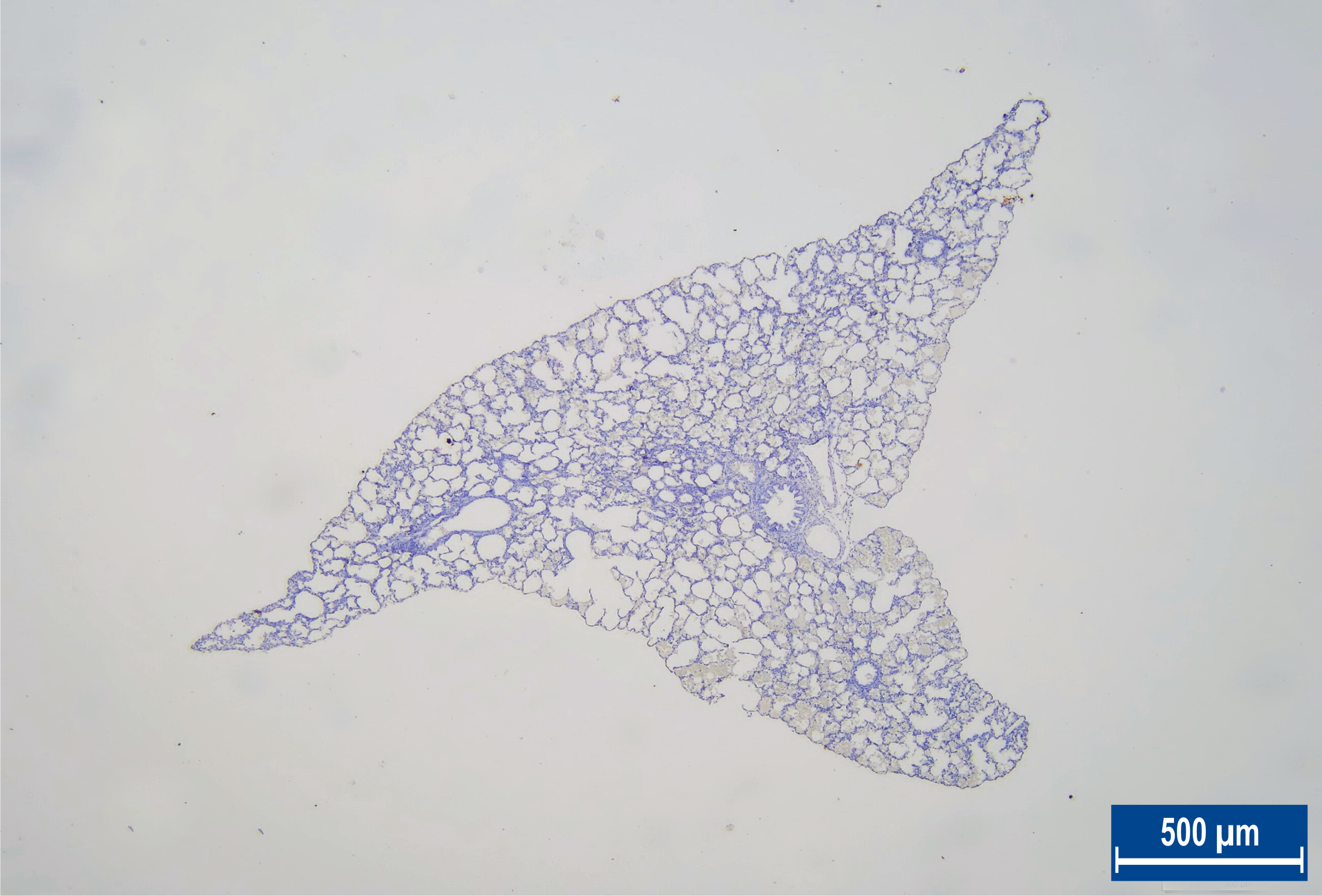

Supplement: S7 Fig — (TIF) [file pone.0320938.s007.tif]
