# Supplementary material for: Extra virgin olive oil mitigates lung injury in necrotizing enterocolitis: Effects on TGFβ1, Caspase-3, and MDA in a neonatal rat model
Source: PLoS One. 2025 Apr 15;20(4):e0320938. doi: 10.1371/journal.pone.0320938 (PMC11999148; doi:10.1371/journal.pone.0320938)
Supplement: Attachment — Submitted filename: Review Report PONE.pdf [file pone.0320938.s009.pdf]

Review Report PONE-D-24-41150: "Extra virgin olive oil mitigates lung injury in necrotizing enterocolitis: effects on TGFβ1, Caspase-3, and MDA in a neonatal rat model"

The manuscript addresses the effects of extra virgin olive oil (EO) on lung injury in a neonatal rat model of necrotizing enterocolitis (NE). The study is well-conceived, providing a novel insight into the therapeutic potential of EO rich in polyphenols in reducing inflammation, oxidative stress, and apoptotic markers such as TGFβ1, MDA, and Caspase-3. The rationale is strong, and the topic is relevant given the lack of effective treatments for NE-associated lung damage in premature infants.

However, some points require clarification, additional data, or reorganization to improve the manuscript's quality. These include methodological details, statistical analysis, and interpretations that need further support.

1. The introduction provides a good background on NE and lung injury but lacks a detailed explanation of why EO was chosen. The manuscript should further elaborate on the specific phenolic compounds in EO that might influence the markers studied (TGFβ1, MDA, Caspase-3). This would help link EO's known biological effects to the expected outcomes more clearly.

2. While the model's basis is well-described, a justification for the number of animals used (n=8 per group) and the sample size calculation is missing. This information is crucial to validate the statistical power of the study.

3. The methods for scoring histopathological damage and evaluating Caspase-3 expression need further detail. For example, it is important to specify how interobserver variability was managed, as scoring can be subjective.

4. While the tables are informative, some figures (e.g., histological images) need improving; low resolution, labels are not clear in some instances and white balance is needed. Without these corrections figures would not proper for publication.

5. The results show a significant reduction in TGFβ1 and MDA levels in the EO group. While this supports EO’s anti-inflammatory and antioxidant effects, further discussion on the physiological mechanisms and how EO’s components interact with pathways related to TGFβ1 and oxidative stress would enrich the analysis.

6. Caspase-3 activity is used as a marker of apoptosis. While the results indicate reduced expression in the EO-treated group, providing data on other apoptotic markers (e.g., Bax, Bcl-2) could offer a more comprehensive view of EO's anti-apoptotic effects.

7. The discussion addresses the findings well but could be strengthened by comparing them with other studies using similar models or interventions (e.g., other antioxidants). Furthermore, the potential limitations of using neonatal rats as a model for human NE should be acknowledged.

8. The conclusion appropriately highlights EO’s therapeutic potential but would benefit from more emphasis on future directions and clinical applicability.

9. The manuscript uses several abbreviations, such as NE, EO, and TGFβ1, without consistently defining them upon first use. Ensuring all abbreviations are clearly defined will improve readability.

10. There are some grammatical and typographical errors throughout the manuscript (e.g., "oragastric" should be "orogastric"). A thorough proofreading would be beneficial.

The manuscript presents valuable findings on the effects of EO on NE-induced lung injury and adds to the literature on potential therapies for NE complications. The authors should address the above points to strengthen the manuscript. With revisions to improve the clarity of the methodology, and the depth of the discussion, the manuscript could be suitable for publication.
