# Supplementary material for: Extra virgin olive oil mitigates lung injury in necrotizing enterocolitis: Effects on TGFβ1, Caspase-3, and MDA in a neonatal rat model
Source: PLoS One. 2025 Apr 15;20(4):e0320938. doi: 10.1371/journal.pone.0320938 (PMC11999148; doi:10.1371/journal.pone.0320938)

PLOS ONE

Extra virgin olive oil mitigates lung injury in necrotizing enterocolitis: effects on TGFβ1, Caspase-3, and MDA in a neonatal rat model

--Manuscript Draft--

**Manuscript Number:**	PONE-D-24-41150
**Article Type:**	Research Article
**Full Title:**	Extra virgin olive oil mitigates lung injury in necrotizing enterocolitis: effects on TGFβ1, Caspase-3, and MDA in a neonatal rat model
**Short Title:**	Effect of extra virgin olive oil on necrotizing enterocolitis -associated lung injury
**Corresponding Author:**	mustafa tuşat<br>Aksaray Üniversitesi Tıp Fakültesi: Aksaray Universitesi Tip Fakultesi<br>aksaray, TÜRKIYE
**Keywords:**	Keywords: necrotizing enterocolitis; lung injury; transforming growt factor beta-1 (TGFß1); extra virgin olive oil; polyphenol
**Abstract:**	Abstract:<br>Background: Necrotizing enterocolitis (NE), which is common in premature babies, has been associated with lung damage. Our aim is to explore the effect of enterally administered extra virgin olive oil (EO) with rich polyphenol content on clinical parameters, histopathological score, Transforming growt factor beta-1 (TGFβ1), Caspase 3 and Malondialdehyde (MDA) levels in NE-related lung injury of neonatal rats.<br>Methods: Three groups (control, NE, NE+EO) were created, with 8 neonatal rats in each group. NE was induced by hypoxia-hyperoxia-hypothermia and formula. EO was given to the treatment group by oragastric probe for 3 days. Intestinal and lung tissue were excised for analysis.<br>Results: TGFβ1 expression levels, TGFβ1 and MDA concentration levels were higher in the NE compared to NE + EO and control groups ($p<0.001$), and their levels decreased after EO treatment compared to the NE group ($p<0.001$). It was determined that EO treatment significantly reduced the histopathological damage and the caspase-3 expression level in the lung ($p<0.001$). Conclusion: Our findings emphasize that TGFß1 has an crucial function in NE-related lung injury and that EO has therapeutic potential in NE-related lung injury.
**Order of Authors:**	Mustafa Tuşat
	Recep Eröz
	Ferhan Bölükbaş
	Erkan Özkan
	Mehmet Semih Demirtaş
	Hüseyin Erdal
	Osman Okan Özocak
**Additional Information:**	
**Question**	**Response**
**Financial Disclosure**<br><br>Enter a financial disclosure statement that describes the sources of funding for the work included in this submission. Review the submission guidelines for detailed requirements. View published research articles from *PLOS ONE* for specific	The author(s) received no specific funding for this work.

examples.<br><br>This statement is required for submission and **will appear in the published article** if the submission is accepted. Please make sure it is accurate.<br><br>**Funded studies**<br>Enter a statement with the following details:<br>• Initials of the authors who received each award<br>• Grant numbers awarded to each author<br>• The full name of each funder<br>• URL of each funder website<br>• Did the sponsors or funders play any role in the study design, data collection and analysis, decision to publish, or preparation of the manuscript?<br><br>Did you receive funding for this work?	
**Competing Interests**<br><br>Use the instructions below to enter a competing interest statement for this submission. On behalf of all authors, disclose any competing interests that could be perceived to bias this work—acknowledging all financial support and any other relevant financial or non-financial competing interests.<br><br>This statement is required for submission and **will appear in the published article** if the submission is accepted. Please make sure it is accurate and that any funding sources listed in your Funding Information later in the submission form are also declared in your Financial Disclosure statement.<br><br>View published research articles from *PLOS ONE* for specific examples.	The authors have declared that no competing interests exist

**NO authors have competing interests**<br><br>Enter: *The authors have declared that no competing interests exist.*<br><br>**Authors with competing interests**<br><br>Enter competing interest details beginning with this statement:<br><br>*I have read the journal's policy and the authors of this manuscript have the following competing interests: [insert competing interests here]*<br><br>* typeset	
**Ethics Statement**<br><br>Enter an ethics statement for this submission. This statement is required if the study involved:<br><br>• Human participants<br>• Human specimens or tissue<br>• Vertebrate animals or cephalopods<br>• Vertebrate embryos or tissues<br>• Field research<br><br>Write "N/A" if the submission does not require an ethics statement.<br><br>General guidance is provided below. Consult the submission guidelines for detailed instructions. **Make sure that all information entered here is included in the Methods section of the manuscript.**	For this study, approval was received from the Animal Experiments Ethics Committee of Aksaray University Experimental Animals Application and Research Center (dated 24/04/2024 and decision number 2024/04-16) and the National Institute of Health's Guide for the Care and Use of Laboratory Animals was followed throughout the experiment. Neonatal rats were sacrificed with high doses of ketamine and xylazine before surgical procedures. Every effort was made to minimize pain during the experimental procedures.

Powered by Editorial Manager® and ProduXion Manager® from Aries Systems Corporation

**Format for specific study types**<br><br>**Human Subject Research (involving human participants and/or tissue)**<br>• Give the name of the institutional review board or ethics committee that approved the study<br>• Include the approval number and/or a statement indicating approval of this research<br>• Indicate the form of consent obtained (written/oral) or the reason that consent was not obtained (e.g. the data were analyzed anonymously)<br><br>**Animal Research (involving vertebrate animals, embryos or tissues)**<br>• Provide the name of the Institutional Animal Care and Use Committee (IACUC) or other relevant ethics board that reviewed the study protocol, and indicate whether they approved this research or granted a formal waiver of ethical approval<br>• Include an approval number if one was obtained<br>• If the study involved *non-human primates*, add *additional details* about animal welfare and steps taken to ameliorate suffering<br>• If anesthesia, euthanasia, or any kind of animal sacrifice is part of the study, include briefly which substances and/or methods were applied<br><br>**Field Research**<br><br>Include the following details if this study involves the collection of plant, animal, or other materials from a natural setting:<br>• Field permit number<br>• Name of the institution or relevant body that granted permission	
**Data Availability**<br><br>Authors are required to make all data underlying the findings described fully available, without restriction, and from the time of publication. PLOS allows rare exceptions to address legal and ethical concerns. See the PLOS Data Policy and FAQ for detailed information.	Yes - all data are fully available without restriction

Powered by Editorial Manager® and ProduXion Manager® from Aries Systems Corporation

A Data Availability Statement describing where the data can be found is required at submission. Your answers to this question constitute the Data Availability Statement and **will be published in the article**, if accepted.<br><br>**Important:** Stating 'data available on request from the author' is not sufficient. If your data are only available upon request, select 'No' for the first question and explain your exceptional situation in the text box.<br><br>Do the authors confirm that all data underlying the findings described in their manuscript are fully available without restriction?	
**Describe where the data may be found in full sentences. If you are copying our sample text, replace any instances of XXX with the appropriate details.**<br><br>• If the data are **held or will be held in a public repository**, include URLs, accession numbers or DOIs. If this information will only be available after acceptance, indicate this by ticking the box below. For example: *All XXX files are available from the XXX database (accession number(s) XXX, XXX.).*<br>• If the data are all contained **within the manuscript and/or Supporting Information files**, enter the following: *All relevant data are within the manuscript and its Supporting Information files.*<br>• If neither of these applies but you are able to provide **details of access elsewhere**, with or without limitations, please do so. For example:<br><br>*Data cannot be shared publicly because of [XXX]. Data are available from the XXX Institutional Data Access / Ethics Committee (contact via XXX) for researchers who meet the criteria for access to confidential data.*<br><br>*The data underlying the results presented in the study are available from (include the name of the third party*	The data underlying the results presented in this study can be obtained from the corresponding author

and contact information or URL).<br>• This text is appropriate if the data are owned by a third party and authors do not have permission to share the data.<br><br>* typeset	
Additional data availability information:	

Manuscript Click here to access/download;Manuscript;manuscript.docx

Extra virgin olive oil mitigates lung injury in necrotizing enterocolitis: effects on *TGFβ1*, Caspase-3, and MDA in a neonatal rat model

Effect of extra virgin olive oil on necrotizing enterocolitis -associated lung injury

Mustafa Tuşat [1, *], Recep Eröz [2], Ferhan Bölükbaş [3], Erkan Özkan[4], Mehmet Semih Demirtaş [5], Hüseyin Erdal [6], Osman Okan Özocak [7]

[1] Aksaray University Medical Faculty, Department of Pediatric Surgery, Aksaray, Turkey; mustafatusat42@hotmail.com

[2] Aksaray University Medical Faculty, Department of Medical Genetics, Aksaray, Turkey; eroz38r@hotmail.com

[3] Aksaray University Veterinary Faculty, Department of Histology and Embryology, Aksaray, Turkey; ferhanbolukbas@gmail.com

[4] Aksaray University Veterinary Faculty, Department of Parasitology, Aksaray, Turkey; erkanozkan87@gmail.com

[5] Aksaray University Medical Faculty, Department of Pediatrics, Aksaray, Turkey; md.semihdemirtas@gmail.com

[6] Aksaray University Medical Faculty, Department of Medical Genetics, Aksaray, Turkey; herdalyfa@gmail.com

[7] Erciyes University Medical Faculty, Department of Cardiovascular Surgery, Kayseri, Turkey; okanozocak@erciyes.edu.tr

* Correspondence: mustafatusat42@hotmail.com; Tel.: 03825022001

Abstract:

Background: Necrotizing enterocolitis (NE), which is common in premature babies, has been associated with lung damage. Our aim is to explore the effect of enterally administered extra virgin olive oil (EO) with rich polyphenol content on clinical parameters, histopathological score, *Transforming growt factor beta-1* (*TGFβ1*), Caspase 3 and Malondialdehyde (MDA) levels in NE-related lung injury of neonatal rats.

Methods: Three groups (control, NE, NE+EO) were created, with 8 neonatal rats in each group. NE was induced by hypoxia-hyperoxia-hypothermia and formula. EO was given to the treatment group by oragastric probe for 3 days. Intestinal and lung tissue were excised for analysis.

Results: *TGFβ1* expression levels, *TGFβ1* and MDA concentration levels were higher in the NE compared to NE + EO and control groups ($p<0.001$), and their levels decreased after EO treatment compared to the NE group ($p<0.001$). It was determined that EO treatment significantly reduced the histopathological damage and the caspase-3 expression level in the lung ($p<0.001$).

Conclusion: Our findings emphasize that *TGFß1* has an crucial function in NE-related lung injury and that EO has therapeutic potential in NE-related lung injury**.**

Keywords: necrotizing enterocolitis; lung injury; *transforming growt factor beta-1* (*TGFß1*); extra virgin olive oil; polyphenol

1. Introduction

Necrotizing enterocolitis (NE) is a life-threatening gastrointestinal problem characterized by inflammation, necrosis and frequently affecting the terminal ileum and colon, seen especially in premature newborns in the presence of various risk and etiological factors [1]. It is known that NE affects many organs [2] and lung damage occurs in 10-15% of premature babies with NE [3]. This lung damage is an important complication of NE, and NE is an important predisposing risk factor for the development of more severe lung damage than broncho pulmonary dysplasia (BPD), which can often be observed in premature babies [2, 4].

Transforming growth factor-beta-1 (*TGFβ1*) is a member of the TGF-β family of growth factors, which has various physiological roles such as apoptosis, inflammation, proliferation, differentiation, and control of immune function, and *TGFβ1* is the *TGF* isoform most frequently formed in tissue damage [5]. It is known that *TGFβ1* plays a role in lung damage in which sepsis, fibrosis, inflammation and epithelial-mesenchymal transformation occur [6, 7]. It has been reported that pro-inflammatory *TGFβ1* cytokine levels are increased in the lungs of babies with neonatal hyaline membrane disease and neonatal BPD [8], and it has been reported that the expression and protein levels of this proinflammatory cytokines (PIC) increase in the bronchoalveolar-lavage fluid (BALF) and lung tissue in acute lung injury (ALI) and bleomycin-induced lung fibrosis [9, 10].

Extra virgin olive oil (EO), fundamental component of the Mediterranean diet, has positive effects on human health mainly caused by the polyphenol content of EO [11]. In studies conducted on humans and animals in vivo or in vitro, EO has beneficial effects resulting from its anti-inflammatory, antioxidant and antimicrobial properties [1, 11, 12]. Enteral administration of tyrosol (Tyr), Oleuropein (Ole) and hydroxytyrosol (HTyr), which are polyphenol compounds of EO, reduces lipopolysaccharide (LPS)-induced ALI by inhibiting inflammation and oxidative stress (OS) [13-16], and the unsaponifiable fractions of orally administered EO reduce aluminum

chloride and acrylamide-induced lung and DNA damage [17], and Ole has an antifibrotic effect by exhibiting anti-inflammatory and antioxidant properties in bleomycin-induced lung fibrosis in rats [18]. Additionally, in our previous study, the protective effect of phenol-rich EO on the intestines of rats with experimental NE was reported by showing anti-inflammatory, antioxidant, anti-apoptotic and cytoprotective effects [1]. Considering the anti-inflammatory, antioxidant and antimicrobial properties of EO and related studies, it is likely that EO has a positive effect on NE-related lung damage. To our knowledge, the effect of EO rich in phenol content on lung damage in rats with NE has not been investigated.

In our study, we aimed to evaluate the impact of enterally administered EO with rich polyphenol content on clinical parameters, histopathological scoring (HPS), caspase-3 positive cells (Casp-3^{+} cells), *TGFβ1* gene expression level and *TGFβ1*, MDA lung tissue homogenate level on lung damage caused by the NE model experimentally created in rats**.**

2. Materials and Methods

2.1 Ethics

For this study, approval was received from the Animal Experiments Ethics Committee of Aksaray University Experimental Animals Application and Research Center (dated 24/04/2024 and decision number 2024/04-16) and the National Institute of Health's Guide for the Care and Use of Laboratory Animals was followed throughout the experiment. Neonatal rats were sacrificed with high doses of ketamine and xylazine before surgical procedures. Every effort was made to minimize pain during the experimental procedures.

2.2 Animals

Neonatal rats used in the study were obtained from Aksaray University Animal Experiments Application and Research Center. For the study, 4 Wistar Albino female rats, 10-12 weeks old

and weighing 200-250 g, were impregnated and kept in a 12-hour light/dark environment, 23 ± 1 ˚C temperature, 55-60% humidity in polycarbonate cages. Pellet feed and drinking water were provided ad libitum. Starting from the 20th day of pregnancy, the rats were transferred to cages and monitored. As birth approaches, the frequency of observation is increased. Births were carried out under the supervision of a veterinarian, and neonatal rats were randomly divided into 3 groups, with 8 members. To avoid the protective effect of breast milk, neonatal rats to be induced necrotizing enterocolitis were taken from their mothers immediately after birth and placed in an incubator with a temperature of 37 ˚C and 65-70% humidity, 12 hours dark and 12 hours light. The health and behavior of neonatal rats were monitored hourly during the experiment. Our research team included a veterinarian and the other researchers were certified in the handling of experimental animals. Induction of necrotizing enterocolitis was performed for 3 days and rats were sacrificed on the morning of day 4. Sacrifice was performed by intraperitoneal administration of 10 mg/kg xylazine and 90 mg/kg ketamine. Acetominophen was prepared to be administered oragastrically at a dose of 200mg/kg in the event that pain signs were observed in rats.

2.3 Experimental Model

Neonatal rats in group 1 (Control) stayed with the mother and were fed with breast milk, and this group was not exposed to any procedures. NE induction to the rats in group 2 (NE) and group 3 (NE+EO) was performed according to the model specified by Güven et al. [19]. For NE induction, neonatal rats were fed with formula consisting of a mixture of 100 cc puppy milk (Baephar-Bogena. BV Sedel. The Netherlands) and 20 g similac 60/40 (Ross-Pediatrics. Columbus. Ohio) by gavage and 0.2 ml each time. The daily dose was increased by 0.1ml according to tolerance. Additionally, neonatal rats were exposed to 100% CO_2 inhalation for 10 minutes, followed by 97% O_2 inhalation for 5 minutes, and cold for 5 minutes at +4°C, twice a day for 3 days. EO, which contains rich polyphenols, was given to neonatal rats in Group 3 at a dose of 2ml/kg via

oral gavage for 3 days. Baby rats in control and NE groups were given distilled water at a dose of 2ml/kg via oral gavage. EO (Sidyma, Muğla, Turkey), which contains rich polyphenols with a polyphenolic content of 832mg/kg, was purchased from the local market and the polyphenolic compound levels and fatty acid levels are stated in Table 1.

Table 1: Content of EO administered to rats

Fatty Acid	(%)	Phenolic Composition	ppm
Oleicacid	71.07	Oleuropeinaglycon (monoaldehyde form)	58
Palmiticacid	14.32	Oleuropeinaglycon (dialdehyde form)	78
Linoleicacid	9.08	Oleocantral +Oleacein (index D1)	469
Steraicacid	2.36	Ligstrosideaglycon (monoaldehyde form)	55
Linolenicacid	0.98	Oleocantral	321
Palmitoleicacid	0.83	Ligstrosideaglycon (dialdehyde form)	172
Arachidicacid	0.45	Oleacein	148
Heptadecenoicacid	0.19	Hydroxytyrosol (mg/kg)	283
Beheric-acid	0.12	Tyrosol	549
Gadoleic-acid	0.34	**Total amount of phenolic compounds (ppm)**	832
Lignoseric-acid	0.11		
Heptadecanoic-acid	0.12		
alfa-Tocopherol (ppm)	605		

Fatty Acid Percentage, Phenolic and Polyphenolic Composition of the EO Administered to Rats

2.4 Humane Endpoints of Work

In order to prevent unnecessary pain, suffering and stress in animals used in research, which are both ethically and scientifically important, the humane endpoint criteria specified in the studies were complied with in our study. The Humane endpoints in our study address the physical and psychological status of the animals, such as general health, behavioral changes, pain, weight loss, loss of appetite, and respiratory distress [20]. In our study, if the threshold values of these criteria were reached, euthanasia was performed with high dose anesthesia. The humanitarian endpoint criteria established in the study were evaluated. These criteria are

1-When severe stress is observed in animals (absence of the sucking reflex)

2-If the rat is in severe pain or suffering (continuous struggle)

3-Significant weight loss observed during the course of the experiment, especially if the animal's weight drops by more than 20%

4- When prolonged severe loss of appetite is observed
5- Complete cessation of movement in the neonatal rat
6- Graying of the whole body due to severe breathing difficulties
7- Severe impairment of heart rate, body temperature, respiratory rate and reflexes

In the present study, humane endpoints were tabulated and neonatal rats were monitored hourly throughout the experiment. On the 3rd day of the study, loss of movement and severe respiratory distress were observed in an animal in the NE group. Since the humane endpoint criterion was reached before the end of the experimental period, the neonatal rat was euthanized with high dose anesthesia 15 minutes after the determination of the humane endpoint criterion under the control of a veterinarian. In our study, there were no rats that died without meeting the euthanasia criteria. The other 23 neonatal rats used in our study completed the experiment without reaching the human endpoint criteria.

2.5 Clinical Illness Score (CIS) and Follow-up

Neonatal rats were weighed every day throughout the experiment with a very sensitive balance, including birth weight (BW) and last day weight (LW). The daily CIS of neonatal rats was evaluated according to the scoring system determined by Zani et al [21]. For this purpose, a blind observer calculated a CIS. Deaths due to NE and other causes were recorded daily throughout the experiment.

2.6 Macroscopic Evalution

On the morning of day 4 of the experimental model, neonatal rats were sacrificed with a high dose of anesthetic drug. After entering the abdomen through a median incision and the thorax through a sternotomy, the abdomen and chest cavity were explored. Macroscopic evaluation was performed according to the method described by Zani et al. and bowel macroscopic score (BMS) was calculated [21]. Then, 3 cm of intestine was excised, including the terminal ileum and

proximal colon. In the lung tissue sample, the lower lobe of the right lung was excised. Half of the samples were reserved for immunohistochemical and histopathological analysis, while the other half were washed with saline and stored at -80 °C for genetic and biochemical investigations

2.7 Detection of TGFβ1 and MDA levels via enzyme-linked immunosorbent assay (ELISA) in Lung Tissues

Lung tissue samples from whole rats were analyzed in duplicate. TGFβ1 lung tissue levels were analysed by ELISA method with Rat *TGFβ1* ELISA Kit [catalog number: ELK2311]. The analysis results were expressed as nanograms/milliliter (ng/mL). The MDA (Malondialdehyde) lung tissue levels were analysed with ELISA method and ELK Biotechnology Rat MDA ELISA Kit [catalog number: ELK8612].

2.8 Histopathologic Evalution of Intestine

Tissue specimens were fixed in 10% formalin and embedded in paraffin blocks. Sections (6 µm thick) were taken from the blocks and stained with the Hematoxylin-eosin [22] method for histopathological examination. The prepared preparations were examined under (Leica DM2500, Leica Microsystems GmbH, Germany) and photographs of the necessary areas were taken with a digital camera (Leica DFC 320). Changes observed as a result of histopathological examination of preparations stained with hematoxylin-eosin were scored between 0 and 4. Scoring was performed according to the method described by Caplan 1994 [23]. NE is indicated by a score of 2 or higher, while severe NE is indicated by a score of 3 or higher.

2.9 Histopathologic Evalution of Lung

Lung tissue specimens taken from newborn rats were fixed in 10% formalin for 24 hr, underwent a series of histological preparations and, were sectioned into 6 µm thick tissue sections using a rotary microtome. The sections were stained with Hematoxylin-eosin staining method [22]. Histopathological evaluations were performed using a modified version of the scale developed by the American Thoracic Society (ATS), Made according to Drucker et.al. 2018. Scores from 0 (normal) to 2 (acute lung injury) are given for six different parameters and added up to obtain a score between 0 and 12; 0 indicates normal lung, and 12 indicates severe acute injury with bleeding [24, 25].

2.10 Immunohistochemical Evalution of Lung

Caspase-3 (STJ97448, St John's Laboratory, 1:200 dilution) were satined immunohistochemically using a sensitive peroxidase-labelled streptavidin-biotin detection system (Ultra Tek HRP Anti-Polyvalent Lab. Thermo Fisher Scientific Inc., UK).

All preparations were examined under a light microscope (Leica DM2500) and then were photographed by a digital camera (Leica DFC 320). In the sections stained with the immunohistochemical method, all Casp-3^+ cells were counted in 6 different randomly selected regions of 10.000 µm^2 at x400 magnification.

2.11 R[illegible]A isolation and c[illegible]A synthesis

RNA was isolated from lung samples via both Hybrid-R (Catalog No: 305-101) and RiboEx (Catalog No: 301-001) isolation kits base on the manufacturer's protocol. cDNA was obtained using A.B.T.™ cDNA Synthesis Kit with Rnase Inh. (High Capacity) cDNA synthesis kit (Catalog No: C03-01-05) via ProFlex thermalcycler. A total 20 µL mastermix (10 µl isolated RNA sample, 0.5 µl RNase Inhibitor, 2 µl Random hexamer, 2 µl 10X Reaction Buffer, 1 µl 20X dNTP mix, 1 µl RTase, 3.5 µl RNase free water was used for cDNA synthesis. cDNA synthesis

condition were at 250C for 10 m in step 1, at 370C for 120 m in step 2, at 850C for 5 m in step 3 and finally at 40C for ~ m.

2.12 *TGFβ1* and *GAPDH* gene expressions by Real-Time qPCR

The following primers were used for the genes expression

Gene Name	Primers	Manufacturer
***TGFβ1*-F**	AGGACCCAGATACTCCCAAG	Oligomer
***TGFβ1*-R**	GTCCCCATACACTGCTTCAC	Oligomer
***GAPDH*-F**	GTGGAGTCCTGGAACTGAAGC	Oligomer
***GAPDH*-R**	AGCACCAATCTGTGATGACAAC	Oligomer

For each cDNA sample, expression of TGF1β and the reference gene (GAPDH) were detected via the Applied Biosystems™ QuantStudio5 Real-Time PCR System. Polymerase chain reactions (PCRs)were performed using Mastermix that include 4 µl cDNA Template, 10 µl 2X MasterMix (with SYBR-Green), 1 µl Forward Primer (10 µM), 1 µl Reverse Primer (10 µM), 3 µl RNase-Free Distilled Water, 1 µl ROX Dye with a final volume of 20 µL.

PCR Cycle conditions were 1 cycle initial denaturation at 95^{0}C for 300 sec, 40 cycles denaturation at 95^{0}C for 15 s and 40 cycles annealing at 60^{0}C for 60 second. The *GAPDH* was used as reference gene and fold Change had been calculated.

2.13 Statistical analysis

The Statistical Package for Social Sciences (IBM Corp., Armonk, NY, USA) 22.0 were used for the data analyze. The distribution of data was detected via Shapiro Wilk test. The descriptive statistic was done. Because the normally distribution of data ($p > 0.05$), One-Way Anova test was used for each group comparisons. Since the variances of the groups were homogeneous, Tukey

HSD test was used. Also polynominal regression test was carried out. The $p < 0.05$ was accepted as statistically significant.

Research manuscripts reporting large datasets that are deposited in a publicly available database should specify where the data have been deposited and provide the relevant accession numbers. If the accession numbers have not yet been obtained at the time of submission, please state that they will be provided during review. They must be provided prior to publication.

Interventionary studies involving animals or humans, and other studies that require ethical approval, must list the authority that provided approval and the corresponding ethical approval code.

3. Results

On the 3rd day of the study, one rat in the NE group showed signs of human endpoint and the neonatal rat was euthanized and subsequent laparotomy revealed necrosis and perforation in the terminal ileum. There were meaningful differences among the groups for *TGFβ1* expression, MDA and *TGFβ1* concentration levels, LW, BMS, and CIS (p<0.05) (Table 2, Fig 1).

Table 2: The mean concentration and expression levels of *TGFβ1*, MDA and clinical parameters of all groups

**Parameters**	**Groups**			
	**Control (M±SD)(mi[illegible]ax)**	**NE (M±SD)(min-max)**	**NE+EO(M±SD)(min-max)**	p
**Birth Weight**	6.125±0.585 (5.2-7.2)	6.075±0.689 (5.4-7.1)	6.05±0.316 (5.7-6.6)	0.963
**LDW**	8.85±0.524 (8.2-9.7)	6.325±0.643 (5.6-7.2)	7.70±0.363 (7.4-8.5)	0.000
**CDS**	-	10.13±1.458 (8-12)	2.38±1.302 (0-4)	0.000
**BM**	-	5.13±0.641(4-6)	1.63±0.518 (1-2)	0.000
***TGFβ1* C. Lev**	166.118±22.899 (128.3-198.6)	474.899±94.859(335.83-585.25)	186.65±20.101 (150.19-205.93)	0.000
***MDA* C. Lev**	110.396±8.226 (103.68-127.26)	216.735±33.703 (183.14-272.72)	114.158±5.184 (105.45-119.31)	0.000
***TGFβ1* Exp Lev**	0.558±0.656 (0.08-1.91)	9.883±2.99 (4.8-14.75)	2.648±3.319 (0.15-8.49)	0.000

The mean concentration and expression levels of TGFβ1, MDA and clinical parameters of all groups

NE: Necrotizing Enterocolitis ***EO***: Extra Virgin Olive Oil **LDW:** Last Day Weight **CDS:** Clinical Disease Score **BM:** Bowel Macroscopy Score ***TGFβ1*:**Transforming growth factor beta 1 ***MDA***: Malondialdehyde **C**: Concentrations **Exp**: Expression **Lev:**Levels ***M***: Mean, ***SD*** : Standard Deviation

Fig 1. Illustrative examples of our study.

Three main groups rats as control, NE, and NE+EO each of them with 8 members were included in the study. Both *TGFβ1*gene expression and concentration levels, MDA concentration levels, histopathological scoring and Caspase-3 positive cell count/unit area of groups were detected. As a response to the cellular damage caused by NE, the expression and translation levels of the different genes may be changed in the nucleus of cells. Depending on the NE, the lung tissue concentration level of MDA significantly increased (a). Also the histopathological scoring (H&E scored 0-12) (b) and Caspase-3 positive cell count/unit area (c) in lung tissue significantly increased depending on the NE injuries. The *TGFβ1*gene is transcribed *TGFβ1* mRNA, and *TGFβ1* proteins are translated from mature *TGFβ1* mRNA. There was statistically significant differences among the groups in terms of *TGFβ1 gene* expression levels. The expression level of *TGFβ1* gene significantly increased depending on the NE injuries (d). Also depending on the NE, the lung tissue concentration level of *TGFβ1* significantly increased (e). It may be said that, the EO has an important protective effect against lung tissue damage caused by NE injuries.

HPS of bowels, Casp-3^{+} cells and HPS for lungs were significantly difference among the goups ($p<0.001$) (Table 3).

Table 3: Caspase-3 Positive Cell Count/Unit Area and Histopathological Scoring of all groups

	Control	NE	NE+EO	p
**HPS for lungs**	0.63±0.744(0-2)	8.63±1.302(7-11)	4±1.195(3-6)	0.000
**Casp-3^{+} cells for lungs**	6.75±2.053(3-9)	36.75±5.994(29-47)	14.88±2.642(11-19)	0.000
**HPS for bowels**	0.0±0.0(0-0)	3.25±0.707(2-4)	1.13±0.641(0-2)	0.000

Caspase-3 Positive Cell Count/Unit Area and Histopathological Scoring of all groups

Casp-3^{+} cells: Caspase-3 Positive Cell Count/unit **HPS:** Histopathological Scoring

The lungs of rats in control group showed normal histology. In NE group, significant histopathological lesions such as neutrophil infiltration, hyperemia, bleeding, and thickening of the interalvelor septum were detected in the lung tissue. It was observed that the histopathological damage to the lung was importantly reduced in the NE+EO group ($p<0.001$) (Table 3,4 Fig 2 A, B, C).

Table 4: Binary comparison of the groups for both laboratory and clinical parameters

	Groups	Control	NE	NE+EO
**Birth Weight**	**Control**	-	0.980	0.960
	**NE**	0982	-	0.985
	**NE+EO**	0.961	0.985	-
		**Control**	**NE**	**NE+EO**
**LDW**	**Control**	-	**0.000**	**0.001**
	**NE**	**0.000**	-	**0.000**
	**NE+EO**	**0.001**	**0.000**	-
		**Control**	**NE**	**NE+EO**
	**Control**	-	**0.000**	**0.001**
	**NE**	**0.000**	-	**0.000**
**CDS**	**NE+EO**	**0.001**	**0.000**	-
		**Control**	**NE**	**NE+EO**
**BM**	**Control**	-	**0.000**	**0.000**
	**NE**	**0.000**	**-**	**0.000**
	**NE+EO**	**0.000**	**0.000**	-
		**Control**	**NE**	**NE+EO**
***TGFβ1* Concentration Levels**	**Control**	-	**0.000**	0.758
	**NE**	**0.000**	-	**0.000**
	**NE+EO**	0.758	**0.000**	-
		**Control**	**NE**	**NE+EO**
***MDA* Concentration Levels**	**Control**	-	**0.000**	0.927
	**NE**	**0.000**	-	**0.000**
	**NE+EO**	0.927	**0.000**	-
		**Control**	**NE**	**NE+EO**
***TGFβ1 E*xpresssion Levels**	**Control**	-	**0.000**	0.266
	**NE**	**0.000**	-	**0.000**
	**NE+EO**	0.266	**0.000**	-
		**Control**	**NE**	**NE+EO**
**HPS for lungs**	**Control**	**-**	**0.000**	**0.000**
	**NE**	**0.000**	**-**	**0.000**
	**NE+EO**	**0.000**	**0.000**	-
		**Control**	**NE**	**NE+EO**
**Casp-3^{+} cells for lung**	**Control**	**-**	**0.000**	**0.001**
	**NE**	**0.000**	**-**	**0.000**
	**NE+EO**	**0.001**	**0.000**	-

Binary comparison of the groups for both laboratory and clinical parameters

NE*:** Necrotizing Enterocolitis ***EO: Extra Virgin Olive Oil ***TGFβ1*:**Transforming growth factor beta-1 ***MDA***: Malondialdehyde **LDW**: Last Day Weight **HPS:** Histopathological Scoring **CDS:** Clinical Disease Score

BM: Bowel Macroscopy Score **Casp-3⁺ cells:** Caspase-3 Positive Cell Count/unit Area Statistical significant p-values are described as **dark color** punto.

Fig 2. Histological evaluation of the lung in neonatal rats from each experimental group.

A. Control group, Bar: 100µm, B. NE group showing increased neutrophil infiltration and severe lung tissue damage, C. treatment group. Haemotoxylene-Eosin staining (H-E) Bar: 50µm. Caspas-3 staining in lung tissue from neonatal rats in all groups; D. Negative control. E. Control group. F. NE group, G. Treatment group. Arrows: Caspase-3 positive cells. It is noteworthy that caspase-3 positive cells were more numerous in the NE group than in the treatment group. Streptavidin-biotin-peroxidase method, Bar: 50µm.

Immunohistochemically, caspase-3 activity was evaluated to detect the apoptosis process in the lung in all groups. It was determined that caspase-3 expressions increased in the lung cells of the NE group. It was observed that caspase-3 expressions decreased significantly in the NE+EO group ($p<0.001$) (Table 3, 4 Fig 2 E, F, G).

According to binary comparison, important differences between NE and NE+EO, between control and both NE and NE+EO were detected in terms of LW, CIS and BMS ($p\leq0.001$), (Table 4).

Also meaningful differences between NE and NE+EO, between control and NE were detected in terms of TGFβ1 Expresssion Levels, Concentration Levels of TGFβ1 and MDA ($p<0.001$), (Table 4).

When the Casp-3^{+} cells and HPS for lungs tobe considered, statistically significant differences between NE and NE+EO, between control and both NE and NE+EO were detected ($p\leq0.001$), (Table 4).

According to polynominal regression analaysis results, meaningful relations were detected between TGFβ1 Concentration levels and all of LW, CIS, BMS, MDA, TGFβ1 Expressions, Casp-3^{+} cells and HPS for lung and ($p<0.001$) (Table 5, Fig 3).

Table 5: Model Summary and Parameter Estimates for both concentration levels of *TGF1β*, MDA and clinical parameters

		Model Summary					Parameter Estimates			
**Variable**	***Equation***	R^2	***F***	***df1***	***df2***	***sig***	**Constant**	***b1***	***b2***	***b3***
TGFβ1 Conc Lev and Birth Weight	Linear	,059	1,388	1	22	,251	6,314	-,001		
	Log	,030	,688	1	22	,416	7,098	-,185		
	Cubic	,308	2,970	3	20	,056	5,011	,008	-7,773	-8,126
TGFβ1 Conc Lev and LDW	Linear	,749	65,611	1	22	,000	9,432	-,007		
	Log	,736	61,302	1	22	,000	18,649	-2,008		
	Cubic	,749	19,935	3	20	,000	9,576	-,008	1,243	3,695
TGFβ1 Conc Lev and CDS	Linear	,918	245,857	1	22	,000	-3,629	,028		
	Log	,930	293,366	1	22	,000	-44,132	8,796		
	Cubic	,938	100,268	3	20	,000	-4,974	,031	3,551	-6,781
TGFβ1 Conc Lev and Bowel Macroscopy	Linear	,861	136,582	1	22	,000	-1,456	,013		
	Log	,891	179,204	1	22	,000	-20,945	4,224		
	Cubic	,896	57,348	3	20	,000	-5,182	,045	-6,460	3,461
TGFβ1 Conc Lev and MDA Conc Lev	*Linear*	,956	482,629	1	22	,000	52,715	,342		
	Log	,911	223,937	1	22	,000	-419,652	103,214		
	Cubic	,961	164,019	3	20	,000	91,725	,001	,001	-4,952
TGFβ1 Conc Lev and Casp-3+ cells for lung	Linear	,928	281,996	1	22	,000	-3,787	,084		
	Log	,934	312,198	1	22	,000	-124,106	26,145		
	Cubic	,938	100,491	3	20	,000	-15,739	,184	,000	1,060
TGFβ1 Conc Lev and HPS for lung	Linear	,797	86,550	1	22	,000	-1,198	,020		
	Log	,823	102,367	1	22	,000	-30,691	6,394		
	Cubic	,824	31,295	3	20	,000	-6,997	,070	,000	6,641
TGFβ1 Conc Lev and TGFβ1 Exp Lev	Linear	,840	115,681	1	22	,000	146,991	29,547		
	Log	,605	33,684	1	22	,000	252,770	65,428		
	Cubic	,868	43,684	3	20	,000	171,175	-5,787	4,943	-,165
MDA Conc Lev and Birth Weight	Linear	,058	1,356	1	22	,257	6,431	-,002		
	Log	,044	1,006	1	22	,327	7,752	-,338		
	Cubic	,177	2,259	2	21	,129	5,663	,000	6,624	-2,621
MDA Conc Lev and LDW	Linear	,764	71,196	1	22	,000	10,407	-,019		
	Log	,783	79,524	1	22	,000	23,204	-3,156		
	Cubic	,779	36,948	2	21	,000	12,148	-,041	6,398	,000
MDA Conc Lev and CDS	Linear	,908	216,507	1	22	,000	-7,650	,080		
	Log	,934	311,018	1	22	,000	-62,119	13,430		
	Cubic	,940	165,323	2	21	,000	-17,716	,210	,000	,000
MDA Conc Lev and Bowel Macroscopy	Linear	,864	140,193	1	22	,000	-3,409	,038		
	Log	,893	183,230	1	22	,000	-29,559	6,445		
	Cubic	,901	95,706	2	21	,000	-8,663	,106	,000	,000
MDA Conc Lev and Casp-3+ cells	Linear	,912	228,681	1	22	,000	-15,677	,239		
	Log	,926	276,983	1	22	,000	-176,362	39,674		
	Cubic	,926	132,102	2	21	,000	-35,358	,493	-,001	,000
MDA Conc Lev and HPS for lung	Linear	,796	86,037	1	22	,000	-4,136	,058		
	Log	,828	105,847	1	22	,000	-43,810	9,771		
	Cubic	,833	52,456	2	21	,000	-12,416	,165	,000	,000
MDA Conc Lev and TGFβ1 Exp Lev	Linear	,805	90,958	1	22	,000	-7,344	,080		
	Log	,806	91,175	1	22	,000	-60,393	13,120		
	Cubic	,807	43,840	2	21	,000	-9,645	,109	-8,459	,000

Model Summary and Parameter Estimates for both concentration levels of *TGF1β*, MDA and clinical parameters

TGFβ1*:**Transforming growth factor beta 1 ***MDA: Malondialdehyde **Conc**: Concentrations **Lev:**Levels **HPS:** Histopathological Scoring **CDS:** Clinical Disease Score **Casp-3⁺ cells:** Caspase-3 Positive Cell Count/unit Area **LDW**: Last Day Weight

Fig 3: Relation between the *TGFβ1* concentration levels in lung tissue and clinical parameters of the groups.

There were statistically significant relation between *TGFβ1* concentration levels and all of last day weight (b), clinical disease score(c), bowel macroscopy(d), MDA concentration levels (e), Caspase-3 positive cell count/unit (f) and histopathological scoring (H&E scored 0-12) (g). Conversly the relation between *TGFβ1* concentration and birth day was not significant (a).

Additionally, there were meaningful relations between MDA and all of LW, CIS, BMS, *TGFβ1* Expressions, $Casp\text{-}3^{+}$ cells and HPS for lung ($p<0.001$) (Table 5, Fig 4).

Fig 4. Relation between the *MDA* concentration levels in lung tissue and clinical parameters of the groups.

There were statistically significant relation between *MDA* concentration levels and all of last day weight (b), clinical disease scor(c), bowel macroscopy(d), Caspase-3 positive cell count/unit (e) and histopathological scoring (H&E scored 0-12) (f). Conversly the relation between MDA concentration and birth day was not significant (a).

When TGFβ1 expression levels to be considered, meaningful relation between *TGFβ1* expression levels and all of LW, CIS, BMS, MDA, $Casp\text{-}3^{+}$ cells, HPS for lung and *TGFβ1* Concentration levels ($p<0.001$) (Table 6, Fig 5).

Table 6: Model Summary and Parameter Estimates for expression levels of *TGFβ1* with all of Caspase 3, MDA and clinical parameters

Model Summery	Parameter Estimates

Variable	*Equation*	R^2	*F*	*df1*	*df2*	*sig*	Constant	*b1*	*b2*	*b3*
TGFβ1 Exp	Linear	,077	1,836	1	22	,189	6,217	-,031		
Lev and Birth	Log	,007	,151	1	22	,702	6,092	-,024		
Weight	Cubic	,272	2,491	3	20	,090	5,991	,307	-,048	,002
TGFβ1 Exp	Linear	,744	63,802	1	22	,000	8,543	-,210		
Lev and LDW	Log	,595	32,346	1	22	,000	7,799	-,491		
	Cubic	,747	19,663	3	20	,000	8,473	-,091	-,020	,001
TGFβ1 Exp	Linear	,862	137,513	1	22	,000	,316	,883		
Lev and CDS	Log	,717	55,620	1	22	,000	3,425	2,100		
	Cubic	,864	42,442	3	20	,000	,390	,647	,053	-,003
TGFβ1 Exp	Linear	,807	92,098	1	22	,000	,421	,419		
Lev and Bowel	Log	,701	51,698	1	22	,000	1,890	1,020		
Macroscopy	Cubic	,811	28,594	3	20	,000	,299	,588	-,022	,001
TGFβ1 Exp	Linear	,805	90,958	1	22	,000	102,955	10,118		
Lev and MDA	Log	,560	27,959	1	22	,000	139,318	22,014		
Conc Lev	Cubic	,844	35,956	3	20	,000	110,388	1,632	,811	-,008
TGFβ1 Exp	Linear	,840	115,681	1	22	,000	146,991	29,547		
Lev and	Log	,605	33,684	1	22	,000	252,770	65,428		
TGFβ1 Conc Lev	Cubic	,868	43,684	3	20	,000	171,175	-5,787	4,943	-,165
TGFβ1 Exp	Linear	,805	90,958	1	22	,000	102,955	10,118		
Lev and	Log	,560	27,959	1	22	,000	139,318	22,014		
Caspase-3⁺ Cells	Cubic	,844	35,956	3	20	,000	110,388	1,632	,811	-,008
TGFβ1 Exp	Linear	,857	131,707	1	22	,000	1,450	,680		
Lev and HPS	Log	,723	57,351	1	22	,000	3,841	1,630		
for lung	Cubic	,860	40,859	3	20	,000	1,313	,842	-,016	,000

Model Summary and Parameter Estimates for expression levels of *TGFβ1* with all of Caspase 3, MDA and clinical parameters

TGFβ1*:**Transforming growth factor beta 1 ***MDA: Malondialdehyde **Conc**: Concentrations **Lev:**Levels **LDW**: Last Day Weight **HPS:** Histopathological Scoring **CDS:** Clinical Disease Score **Casp-3$^+$ cells:** Caspase-3 Positive Cell Count/unit Area

Fig 5. Relation between the *TGFβ1* expression levels in lung tissue and clinical parameters of the groups.

There were statistically significant relation between *TGFβ1* expression levels and all of last day weight (b), clinical disease scor(c), bowel macroscopy(d), MDA concentration levels (e), Caspase-3 positive cell count/unit (f), histopathological scoring (H&E scored 0-12) (g) and *TGFβ1* concentration levels (h). Conversly the relation between *TGFβ1* expression levels iin lung tissue and birth day was not significant (a).

There were statistically significant relation between *TGFβ1* expression levels and all of last day weight (b), clinical disease scor(c), bowel macroscopy(d), MDA concentration levels (e),

Caspase-3 positive cell count/unit (f), histopathological scoring (H&E scored 0-12) (g) and *TGFβ1* concentration levels (h). Conversly the relation between *TGFβ1* expression levels iin lung tissue and birth day was not significant (a).

4. Discussion

In lung injury, various studies have been conducted about the role of genetic markers such as *nuclear factor kappa-B (NF-κB), CASP8, MA[illegible]K[illegible], CASP3, B[illegible]in-1, P[illegible]C and rD[illegible]A* genes for maintaining the cellular homeostasis and viability [26-30].

To our knowledge, our study is the first that showed EO with rich phenol content administered by gavage in the hypoxia-hypothermia-hyperosmolar food-induced NE model of rats, which significantly reduces *TGFß1* and MDA tissue homogenate levels, expression level of *TGFß1* gene and tissue damage in the lung tissue ($p<0.05$).

In our study, it was observed that CIS, BMS and histopathological scores were importantly higher in NE induction group than in healthy group, and LW of the rats were significantly lower than control group. In addition, in the HPS of the intestine, one rat received 2 points, four rats received 3 points and three rats received 4 points, and in line with these data, we concluded the adequacy of the model in creating NE.

In previous studies investigating NE-related lung injury, it was reported that NE causes an increase in inflammation in the lungs, neutrophil infiltration, significant histopathological changes such as thickening and bleeding in the alveolar septum [2, 4, 25, 31-33], and an increase in apoptosis [2, 31, 32]. In our study, significant histopathological lesions such as neutrophil infiltration, hyperemia, bleeding, and thickening of the interalvelor septum were detected in the lung tissue of NE group. It was observed that histopathological scores and Casp-3^+ cells numbers in the lung tissue were significantly decreased in the NE+ EO group compared to the NE group ($p<0.001$, $p<0.001$ respectively). We determined that enteral EO administration reduced

apoptosis by reducing Casp-3^{+} cells in the lung tissue and had a histological protective effect on NE-related lung damage.

Olive oil (olivea oleum), an important part of the Mediterranean diet, is grown throughout the Mediterranean basin and is obtained from olives [34]. EO is obtained by mechanical pressing of olives and contains relatively high amounts of polyphenolic [35]. The main components of EO are fatty acids (95-99%), of which 55-83% are monounsaturated fatty acids (MUFA) (especially oleic acid), 4-20% are polyunsaturated fatty acids and 8-14% are fatty acids. The remaining part is minor polar compounds and these compounds constitute 1-2% of EO and include tocopherols, phytosterols, squalene and phenolic compounds with important biological activities [36]. In EO, phenolic compounds are generally found in concentrations ranging from 50 to 940 mg/kg [37]. EO contains at least 36 different phenolics such as HTyr, Tyr, Ole, Ole aglycone, oleocanthral, flavonoids and lignans. The number and concentration of these compounds depend on the variety and maturity of the olive, the age of the tree, the region where it is grown, the time of harvest, the method of production and storage conditions. While refining EO by exposing it to chemical processes does not cause any change in its fatty acid components, it causes a decrease or even disappearance in the amount of phenolic compounds in it [35, 36]. It has been shown that these phenolic compounds, which are responsible for the main biological properties of EO, have effects such as antioxidant [1, 38-40], anti-inflammatory [1, 39, 40], antimicrobial [41], neuroprotective [42], anti-apoptotic [1, 40] and antidysbiotic [43].

Inflammation and OS has crucial role in the pathogenesis of lung injury, and infections, sepsis, environmental pollutants, smoking, allergens and genetic predisposition are among the agents that cause lung damage [44]. Since EO and phenolic compounds have the above-mentioned properties, EO and phenolic compounds have the potential to prevent lung damage by suppressing excessive amounts of PIC and OS in the pathogenesis of lung damage. IL-17A affects OS, DNA damage and apoptosis in the A549 human epithelial cell line in vitro. But OLE reverses these

effects [45]. In a study examining the effects of enterally administered Ole and silver nanoparticle-loaded Ole on lung tissue for 11 days on intraperitoneal lipopolysaccharide (LPS)-induced ALI in albino rats, a significant decrease in MDA occurred in both treatment groups. It was observed that it caused a significant increase in Glutathione (GSH) and superoxide dismutase (SOD) levels. There was a important decrease in the levels of PIC (Tumor necrosis factor-alpha (TNF-α), IL-6 and IL-1β) in the rats given Ole treatment. In the silver-loaded Ole treatment, these PIC were higher than in the Ole-only treated group, but were significantly reduced compared to the LPS induction rats. In addition, *P2X purinoceptor 7 (P2X7R), bax, IL-1β, TNF-α, and TLR4* gene expression was importantly higher in LPS-induced rats, and the expression of these genes was significantly decreased in both treatment groups, and it has been reported that the histological appearances in the lung are similar and that both Ole and silver-loaded Ole reduce OS, inflammation, tissue damage and apoptosis [46] in the control and both treatment groups.

In ALI, which occurred after intratracheal LPS administration to rats for 20 days, it was observed that neutrophils in the BALF increased significantly, MDA levels increased in serum and lung tissue, and GSH levels, GSH-Px and CAT activities decreased. In addition, a significant increase in IL-6, TNF-α, myeloperoxidase (MPO) and NF-kB was observed in BALF, serum and lung tissue, and the interalveolar septum widened due to capillary hyperemia, edema and PMNL infiltration in the lungs after LPS induction. It has been observed that vascular-bronchial interstitial edema, congestion and intense inflammatory cell accumulation occur. Oral administration of Ole simultaneously with LPS induction positively affects the above-mentioned biochemical and pathological changes in serum, BALF and lung tissue, and as a result, Ole causes significant improvements in the histological damage of the lung by reducing OS and inflammation [14]. In a study examining the effect of HTyr, one of the phenolic compounds of EO, on lung damage caused by nasal LPS induction, it was found that HTyr reduced lung edema, inflammatory cells in BALF and lung tissue, and strongly regulates PIC (IL-10, IL-1β, TNF-α,

IL-6) as well as LPS-stimulated sirtuin (SIRT) expression and autophagy inhibition, MAPK phosphorylation is regulated by HTyr and ameliorates lung injury [15]. Exposure to 85dB noise and toluene inhalation for 6 weeks caused lung damage in rats, increased inflammation and OS, and olive extract given orally once a day for 6 weeks reduced MDA levels, catalase. It has been reported that it increases SOD activity and prevents lung damage by suppressing OS and inflammation by reducing PIC such as TNF-α and IL-1β [47]. In the study evaluating the effect of Tyr, one of the important phenolic compounds of EO, on ALI caused by LPS induction in mice, it was found that Tyr reduced PIC (IL-1ß, IL-6, TNF-α) which increased in BALF and lung tissue as a result of LPS induction. It has been reported that the activation of inflammatory molecules such as increased cyclooxygenase (COX)-2, Inducible nitrite oxide synthase (iNOS) and phosphorylated-IκBα is suppressed by the presence of Tyr in lung tissue. The increase in MPO and the decrease in SOD enzymes caused by LPS were inhibited by Tyr. In addition, Tyr reduces the expressions of iNOS, COX-2 and PIC and the nuclear translacion of NF-kB in LPS-stimulated RAW 264.7 macrophage, resulting in Tyr ameliorating LPS-related ALI, inhibiting NF-κB, reducing the release of PIC and it may be a potential therapeutic agent in inflammatory lung diseases [13]. In another study investigating the effect of Try on another LPS-induced mouse lung injury model, it was observed that Tyr improved pulmonary permeability and histopathological changes, decreased the expression of PIC and increased the expression of antioxidant enzymes [16]. Pulmonary fibrosis is a response to endothelial and epithelial cells that are frequently damaged by the invasion of inflammatory cells. In the rat study investigating the effects of Ole on pulmonary fibrosis induced by intratracheal bleomycin, TNF-α, IL-13, TGFβ1, Platelet-derived growth factor (PDFG), lung collagen index and MDA increased significantly in BALF in the bleomycin-administered group, but it has been reported that Ole prevents pulmonary fibrosis by reversing these effects [18].

NE is one of the leading causes of gastrointestinal-related mortality in premature babies [48], and incomplete development of the intestinal mucosal barrier and immune system due to prematurity, increased OS as a result of inadequate antioxidant capacity, dysbiosis, and feeding with osmolar formulas are among the most important predisposing risk factors for NE [49]. While the severity of NE in the intestine is an important determinant of initial mortality in preterms with NE, long-term complications due to NE depend on the severity of other organ involvement, especially the lungs [50]. In particular, NE-related lung damage is more severe and more difficult to treat than BPD, which is frequently observed in the premature neonatal period [2, 4]. Just like NE, the pathogenesis of NE-induced lung damage remains unknown, but current ongoing research focuses on explaining and preventing the mechanisms that lead to NE-related lung damage, which is an important cause of mortality and complication, in addition to studies on the etiopathogenesis and prevention of NE. In a study evaluating NE-related lung injury in mice and the effect of formula enriched with digested fat, NE induction resulted in increased TNF-α and *lipocalin-2* (*Lcn-2*) gene expressions and 3'-nitrotyrosine (3'-NT), an indicator of OS, and the transcription of the superoxide-producing enzyme NADPH oxidase 2 (NOX2) in lung tissue. Histologically, alveolar destruction, vascular extravasation and polymorpho n[illegible]ear leukocyte (PMNL) accumulation occur in the lung, and immunohistologically, MPO activity, the expression of neutrophil-specific enzyme ELANE and *pro-apoptosis gene p53 up-regulated modulator of apoptosis* (*PUMA*) gene are increased and intense apoptosis has been reported to be observed. In addition, it was observed that the building block proteins SP-A and SP-D of surfactant secreted by epithelial type 2 cells, which are an important structure in the defense of the lung, were significantly reduced by immunostaining method. In addition, it has been reported that ZO-1, a tight junction protein in lung tissue, causes a significant decrease in immunostaining and quantity [31]. In a study investigating NE-related lung damage in mice with NE induction and the effect of H2S-Mesalamine, a hydrogen sulfide (H2S) donor, on lung damage, NE induction significantly

increase TLR4 and IL-6 levels in the intestinal and lung tissues of both wild-type and transgenically endothelial nitric oxide synthase (eNOS) gene knockout mice. It was observed that HS derivative treatment attenuated the increase of TLR4 and IL-6 in wild-type mice and induced clinical and histological improvement, but did not have these effects in transgenic mice. As a result, it has been reported that eNOS plays an important role in NE-related lung damage and that H2S-Mesalamine acts through eNOS [33]. In another study, comparing the NE model created in eNOS knockout mice and wild-type mice, it was shown that the lung and intestinal damage in transgenic mice was more severe, and it was reported that eNOS was due to vasodilatation and antioxidant effect nitrite oxide (NO) synthesis in the mesentery [25]. In another experimental NE study, it was reported that NE-related damage occurred in the lungs due to chemotaxis of pro-inflammatory neutrophils to the lungs [4]. In another study, it was reported that the transfer of CD4+ T lymphocytes isolated from the lungs of NE model mice caused lung damage in immunocompromised mice, and that T lymphocytes may have an important role in the damage occurring in the lungs as a result of NE [51]. It has been reported that lung injury occurring in another experimental NE model is associated with Nod-like receptor pyrin domain-containing 3 (NLRP3) inflammasome gene expression and NF-κB pathway activation [32]. In summary, it is predicted that preventing the inflammatory response, OS and chemotaxis of inflammatory cells to the lungs may prevent NE-related lung damage. OS, inflammation, structural and functional factors, and apoptosis appear to be involved in the pathogenesis of NE-associated lung injury.

In our study important differences were detected among the groups for MDA lung tissue concentration levels ($p<0.05$). Immunohistochemically, caspase-3 activity was evaluated to detect the apoptosis process in the lung in all groups. It was determined that caspase-3 expressions increased in the lung cells of the NE group. It was observed that caspase-3 expressions decreased significantly in the NE+EO group ($p<0.001$) (Table 3, 4 Figure 2 E,F,G). Also, statistically

meaningful relation between MDA levels and all of LW, CIS, BMS, Casp-3$^+$ cells and HPS for lung were detected.

TGFβ1 is a pleiotropic cytokine that plays a role in many physiological and pathological processes such as cellular differentiation, migration, apoptosis and regulation of immune systems, immune tolerance and hemostasis, fibrosis, inflammation, and cancer progression, and its effects depend on the tissue and agent [52]. Its opposing effects, both suppressing and activating inflammation, are important in maintaining immunological balance [53]. *TGFβ1* promotes healing by inhibiting the immune response and uncontrolled inflammation. However, *TGFβ1* serves as one of the main mediators and initiates fibrosis and apoptosis endothelial cells that can cause tissue damage if left uncontrolled, and in infective cases the role of *TGFβ1* is largely tissue and pathogen dependent [54]. *TGFβ1* exerts its effects through the SMAD/*TGFβ1* cascade. SMAD proteins are nuclear proteins in TGF-β signaling. In SMAD/*TGFß1* signaling, TGFß1 first binds to TßR-1 and TßR-2 receptors and then TßR-1 phosphorylates SMAD2 and SMAD3 proteins, enabling the activation of these structures. Activated SMAD2 and SMAD3 proteins form a complex with SMAD4 and translocate to the nucleus to regulate transcription in genes where *TGFβ1* is effective [55]. Although the pathogenesis of *TGFβ1*-mediated lung damage is not fully known, studies have reported that *TGFβ1* causes damage to the lungs by increasing lung microvascular and alveolar permeability and increasing actin stress fiber formation [56]. Many studies have been conducted on *TGFβ1*, and in these studies, various pathways that are affected by *TGFβ1* and/or are affected by *TGFβ1* have been stated. In ALI induced in mice after nickel inhalation, a decrease in the expression of genes that play important roles in alveolar fluid resorption, synthesis or reuse of surfactant proteins and phospholipids, and a significant increase in *TGFβ1* levels and gene expression levels in BALF were found. As a result, *TGFβ1* is a central mediator of ALI [57]. While miR-1258 expression and SOD levels were decreased, TGFβ1 expression, p-SMAD3 protein levels, histological changes indicating ALI, IL-6, IL-1β, TNF-α,

MDA level, *Pknox1* gene expression and protein levels were increased in septic ALI patients, LPS-induced BEAS-2B cells and mice. *miR-1258* expression treatment is an effective agent in lung injury by improving the above-mentioned parameters related to inflammation, OS and apoptosis in LPS-induced mouse and BEAS-2B cells, and *miR-1258* expression treatment inhibit inflammation and OS in ALI through the *TGFβ1*/SMAD3 cascade regulated by *Pknox1* [7]. It has been reported that *TGFβ1* expression increases in fibrosis human lung and bleomycin-induced mouse lung tissues. In this study, *TGFβ1* increased the expression of ornithine aminotransferase (OAT) in fibroblasts, and the increase in OAT expression regulated both *TGFβ1*-induced and constitutive expression of collagen, fibronectin and alpha-smooth muscle actin (α-SMA), which are important components of fibrosis, and OAT is important in lung fibrogenesis. It has also been shown that OAT causes OS as a result of the production of Reactive oxygen species (ROS) in the mitochondria by activating proline dehydrogenase, and OAT inhibition with L-canaline inhibits *TGFβ1* activity and signaling pathway. As a result, it was observed that OAT could regulate *TGFβ1* expression and that OAT inhibition inhibited the Smad-dependent and Smad-independent signaling pathways induced by *TGFβ1* [10]. Severe COVID-19 disease is characterized by excessive release of PIC leading to acute respiratory distress syndrome (ARDS) and potentially life-threatening systemic inflammation. In ARDS patients, TGFβ1 increases in the parenchyma and blood in the early stages, and in the later stage, chronic inflammation dominated by *TGFβ* signaling and IgA2 production develops, which induces fibrosis [58]. In a study comparing patient and healthy control groups to investigate whether TGFβ1 has an effect on predicting the severity of the disease in COVID-19, a significant increase in *TGFβ1*, CRP and D-Dimer levels and a positive correlation between *TGFβ1* and CRP were detected in Covid-19 patients compared to the control group. As a result, it has been reported that *TGFβ1* levels are the main molecule for the pathophysiology of the disease and are a marker that can be used in the early diagnosis of COVID-19 and the course of the disease [59]. It has been

reported that TGFß1, which is a predisposing risk factor for the severity of COVID-19, has a profibrotic effect [60] and that TGFß1 is associated with disease severity. In a study, serum *TGFβ1* levels showed a positive correlation with inflammatory markers such as CRP, leukocyte count, absolute neutrophil count, platelet count, fibrinogen in all patients. In this study, higher serum *TGFβ1* concentrations, which increased with the severity of COVID-19, were measured and *TGFβ1* levels at hospitalization is distinctive in predicting the severity and development of complications.

Considering other studies, the role of *TGFβ1* in COVID-19 disease has not been fully determined and conflicting results have been reported in other studies. In one study, high *TGFβ1* levels were detected in COVID-19 patients and *TGFβ1* levels were associated with disease severity [61], in another study, low *TGFβ1* levels were associated with the severity and complications of COVID-19 [62], and in another cross-sectional study, there is no difference in *TGFβ1* levels in COVID-19 and healthy control groups [63]. It can be thought that this situation is due to patient selection, comorbidities and the time of collection of serum samples. Growth and differentiation factors such as Fibroblast growth factor, Platelet derived growth factor (PDGF), Hepatocyte growth factor (HGF), *TGFβ1*, Notch and Wnt and Notch proteins can regulate or induce endothelial-mesenchymal transition and resulting fibrosis, but the severity of fibrosis observed as a result of *TGFβ1* induction is considered the most important agent in the pathogenesis of epithelial-mesenchymal transition and fibrosis due to reasons such as myofibroblast cell induction [64]. In a study evaluating the effect of olive extract on the epithelial-mesenchymal transition mechanism involved in upper respiratory tract diseases, induction of isolated respiratory tract epithelial cells with *TGFβ1* caused a decreased proliferation rate in the cells, a decrease in the expression of E-cadherin, an epithelial protein marker, and an increased expression of vimentin, a mesenchymal marker. It was stated that epithelial-mesenchymal transition was induced, but olive extract inhibited this induction.

As a result, it was reported that olive extract was effective in pathological tissue remodeling and inflammation [65]. In the study investigating the effect of Tyr, one of the phenolics of EO, on the nonalcoholic steatohepatitis (NASH) model, steatosis and hepatic fibrosis and increased *TGFβ1* gene expression were observed. Tyr reversed these effects and had a positive effect on NASH [60]. In a study investigating the effectiveness of HTyr in the renal hypoxia model created by exposing the human proximal tubule cell epithelial cell line (HK-2) to CoCl2 for 24 hours in vitro, HTyr treatment reduced the formation of both ROS and reactive nitrogen species (RNS) and prevented GSH depletion in hypoxia-induced cells. It has also been reported that HTyr has antioxidant, anti-inflammatory and anti-fibrotic effects on hypoxic renal cells by reducing the gene expressions of IL-6 and *TGFß1*, which are modulators of inflammation and fibrosis [66]. Autosomal dominant polycystic kidney disease is a genetic disease that destroys the kidney parenchyma with fluid-filled cysts that lead to kidney failure. The pathological feature of this genetic disease is the development of interstitial inflammation and fibrosis with the accumulation of inflammatory cells. In an in vitro autosomal dominant polycystic kidney disease model, phenolic-rich olive leaf extract deccreased the gene expressions of PIC (IL1ß, IL-6, TNF-α), which play a role in cyst formation, and TGFß1, which has a fibrotic effect, fibronectin, eCadherin and Alpha Smooth Muscle. It has been reported to reduce Actin (αSMA). In a separate series of experiments, it was reported that TGFß1 stimulation of cells has been reported to produce a fibrotic effect and that olive leaf extract had an anti-fibrotic effect by reducing TGFβ1 [67].

According to the our results, there were statistically important differences among the groups for both *TGFβ1* expression and *TGFβ1* concentraion levels were detected. Additionally the differences between NE and NE+EO, between control and NE were significant for *TGFβ1* Expresssion, *TGFβ1* and MDA Concentration Levels ($p<0.001$), There were significant relation between *TGFβ1* levels and all of LW, CIS, BMS, MDA levels, *TGFβ1* Expressions, Casp-3$^+$ cells

and HPS for lung and ($p<0.001$). Also the relation between MDA levels and all of LW, CIS, BMS, *TGFβ1* Expressions, Casp-3$^+$ cells and HPS for lung were significant for lung ($p<0.001$).

5. Conclusions

Our findings emphasize that *TGFß1* has an important role in NEC-related lung injury. It may be said that, the EO has an important protective effect and has therapeutic potential against lung tissue damage caused by NEC injuries.

References

1. Tusat M, Eroz R, Bolukbas F, Ozkan E, Erdal H. Evaluation of the protective and therapeutic effects of extra virgin olive oil rich in phenol in experimental model of neonatal necrotizing enterocolitis by clinical disease score, inflammation, apoptosis, and oxidative stress markers. Pediatr Surg Int. 2024;40(1):80. Epub 20240317. doi: 10.1007/s00383-024-05669-1. PubMed PMID: 38493431.
2. Wang F-S, Yu M-L, Li W-Z, Hong K, Xu C-B, Wang G-H. Intestinal tract and parenteral multi-organ sequential pathological injury caused by necrotizing enterocolitis. BMC pediatrics. 2020;20:1-6.
3. Ganapathy V, Hay JW, Kim JH, Lee ML, Rechtman DJ. Long term healthcare costs of infants who survived neonatal necrotizing enterocolitis: a retrospective longitudinal study among infants enrolled in Texas Medicaid. BMC pediatrics. 2013;13:1-11.
4. Jia H, Sodhi CP, Yamaguchi Y, Lu P, Martin LY, Good M, et al. Pulmonary epithelial TLR4 activation leads to lung injury in neonatal necrotizing enterocolitis. The Journal of Immunology. 2016;197(3):859-71.
5. Yero A, Bouassa R-SM, Ancuta P, Estaquier J, Jenabian M-A. Immuno-metabolic control of the balance between Th17-polarized and regulatory T-cells during HIV infection. Cytokine & Growth Factor Reviews. 2023;69:1-13.
6. Ma K, Li C, Xu J, Ren F, Xu X, Liu C, et al. LncRNA Gm16410 regulates PM2. 5-induced lung endothelial-mesenchymal transition via the TGF-β1/Smad3/p-Smad3 pathway. Ecotoxicology and environmental safety. 2020;205:111327.
7. Xu X, Xu X, Cao J, Ruan L. MicroRNA-1258 suppresses oxidative stress and inflammation in septic acute lung injury through the Pknox1-regulated TGF-β1/SMAD3 cascade. Clinics. 2024;79:100354.
8. Lecart C, Cayabyab R, Buckley S, Morrison J, Kwong K, Warburton D, et al. Bioactive transforming growth factor-beta in the lungs of extremely low birthweight neonates predicts the need for home oxygen supplementation. Neonatology. 2000;77(4):217-23.
9. Fahy RJ, Lichtenberger F, McKeegan CB, Nuovo GJ, Marsh CB, Wewers MD. The acute respiratory distress syndrome: a role for transforming growth factor-β1. American journal of respiratory cell and molecular biology. 2003;28(4):499-503.

10. Lee J-U, Song KS, Hong J, Shin H, Park E, Baek J, et al. Role of lung ornithine aminotransferase in idiopathic pulmonary fibrosis: regulation of mitochondrial ROS generation and TGF-β1 activity. Experimental & Molecular Medicine. 2024;56(2):478-90.
11. Cicerale S, Lucas L, Keast R. Biological activities of phenolic compounds present in virgin olive oil. International journal of molecular sciences. 2010;11(2):458-79.
12. Cicerale S, Lucas L, Keast R. Antimicrobial, antioxidant and anti-inflammatory phenolic activities in extra virgin olive oil. Current opinion in biotechnology. 2012;23(2):129-35.
13. Kim Y-Y, Lee S, Kim M-J, Kang B-C, Dhakal H, Choi Y-A, et al. Tyrosol attenuates lipopolysaccharide-induced acute lung injury by inhibiting the inflammatory response and maintaining the alveolar capillary barrier. Food and Chemical Toxicology. 2017;109:526-33.
14. Dikmen N, Cellat M, Etyemez M, İşler CT, Uyar A, Aydın T, et al. Ameliorative effects of oleuropein on lipopolysaccharide-induced acute lung injury model in rats. Inflammation. 2021;44(6):2246-59.
15. Yang X, Jing T, Li Y, He Y, Zhang W, Wang B, et al. Hydroxytyrosol attenuates LPS-induced acute lung injury in mice by regulating autophagy and sirtuin expression. Current Molecular Medicine. 2017;17(2):149-59.
16. Wang W-c, Xia Y-m, Yang B, Su X-n, Chen J-k, Li W, et al. Protective effects of tyrosol against LPS-induced acute lung injury via inhibiting NF-κB and AP-1 activation and activating the HO-1/Nrf2 pathways. Biological and Pharmaceutical Bulletin. 2017;40(5):583-93.
17. Ghorbel I, Chaâbane M, Boudawara O, Kamoun NG, Boudawara T, Zeghal N. Dietary unsaponifiable fraction of extra virgin olive oil supplementation attenuates lung injury and DNA damage of rats co-exposed to aluminum and acrylamide. Environmental Science and Pollution Research. 2016;23:19397-408.
18. Khalili HR, Adeli Behrooz HR, Rashidi Nooshabadi MR, Geravandi S, Mohammadi MJ, Foruozandeh H. Evaluation of potential anti-fibrotic effect of oleuropein on bleomycin-induced pulmonary fibrosis in rat. Toxin Reviews. 2020;39(2):188-96.
19. Guven A, Gundogdu G, Vurucu S, Uysal B, Oztas E, Ozturk H, et al. Medical ozone therapy reduces oxidative stress and intestinal damage in an experimental model of necrotizing enterocolitis in neonatal rats. Journal of pediatric surgery. 2009;44(9):1730-5.
20. Morton DB, Hau J. Welfare assessment and humane endpoints. Handbook of Laboratory Animal Science: CRC Press; 2021. p. 123-54.
21. Zani A, Cordischi L, Cananzi M, De Coppi P, Smith V, Eaton S, et al. Assessment of a neonatal rat model of necrotizing enterocolitis. European journal of pediatric surgery. 2008;18(06):423-6.
22. Culling CFA, Allison R, Barr W. Cellular pathology technique: Elsevier; 2014.
23. Caplan MS, Hedlund E, Adler L, Hsueh W. Role of asphyxia and feeding in a neonatal rat model of necrotizing enterocolitis. Pediatric pathology. 1994;14(6):1017-28.
24. Matute-Bello G, Downey G, Moore BB, Groshong SD, Matthay MA, Slutsky AS, et al. An official American Thoracic Society workshop report: features and measurements of experimental acute lung injury in animals. American journal of respiratory cell and molecular biology. 2011;44(5):725-38.
25. Drucker NA, Jensen AR, Te Winkel JP, Ferkowicz MJ, Markel TA. Loss of endothelial nitric oxide synthase exacerbates intestinal and lung injury in experimental necrotizing enterocolitis. Journal of pediatric surgery. 2018;53(6):1208-14.
26. Acat M, Yıldız Gülhan P, Eröz R, Ertınmaz Özkan A, Koca O, Çınar C. Evaluation of both expression and serum protein levels of caspase-8 and mitogen-activated protein kinase 1 genes in patients with different severities of COVID-19 infection. Molecular Biology Reports. 2023;50(4):3241-8.

27. Yildiz Gulhan P, Eroz R, Ataoglu O, Ince N, Davran F, Öztürk CE, et al. The evaluation of both the expression and serum protein levels of Caspase-3 gene in patients with different degrees of SARS-CoV2 infection. Journal of Medical Virology. 2022;94(3):897-905.
28. Yesildag K, Eroz R, Genc A, Dogan T, Satici E. Evaluation of the protective effects of morin against acrylamide-induced lung toxicity by biomarkers of oxidative stress, inflammation, apoptosis, and autophagy. Journal of Food Biochemistry. 2022;46(7):e14111.
29. Turan Sönmez F, Eröz R. The role of argyrophilic nucleolar organizing region-associated proteins in clinical exacerbation of chronic obstructive pulmonary disease. Journal of International Medical Research. 2018;46(12):4995-5003.
30. Çolakoğlu S, Saritas A, Eroz R, Oktay M, Yaykasli K, Akoz A, et al. Is one-time carbon monoxide intoxication harmless? Evaluation by argyrophilic nucleolar-organizing regions staining method. Human & experimental toxicology. 2015;34(1):24-31.
31. Sodhi CP, Salazar AJG, Kovler ML, Fulton WB, Yamaguchi Y, Ishiyama A, et al. The administration of a pre-digested fat-enriched formula prevents necrotising enterocolitis-induced lung injury in mice. British Journal of Nutrition. 2022;128(6):1050-63.
32. Filler R, Yeganeh M, Li B, Lee C, Alganabi M, Hock A, et al. Bovine milk-derived exosomes attenuate NLRP3 inflammasome and NF-κB signaling in the lung during neonatal necrotizing enterocolitis. Pediatric Surgery International. 2023;39(1):211.
33. Hosfield BD, Hunter CE, Li H, Drucker NA, Pecoraro AR, Manohar K, et al. A hydrogen-sulfide derivative of mesalamine reduces the severity of intestinal and lung injury in necrotizing enterocolitis through endothelial nitric oxide synthase. American Journal of Physiology-Regulatory, Integrative and Comparative Physiology. 2022;323(4):R422-R31.
34. Waterman E, Lockwood B. Active components and clinical applications of olive oil. Alternative medicine review. 2007;12(4).
35. Cicerale S, Conlan XA, Sinclair AJ, Keast RS. Chemistry and health of olive oil phenolics. Critical reviews in food science and nutrition. 2008;49(3):218-36.
36. Romani A, Ieri F, Urciuoli S, Noce A, Marrone G, Nediani C, et al. Health effects of phenolic compounds found in extra-virgin olive oil, by-products, and leaf of Olea europaea L. Nutrients. 2019;11(8):1776.
37. Servili M, Sordini B, Esposto S, Urbani S, Veneziani G, Maio ID, et al. Biological activities of phenolic compounds of extra virgin olive oil. Antioxidants. 2013;3(1):1-23.
38. De Bruno A, Romeo R, Piscopo A, Poiana M. Antioxidant quantification in different portions obtained during olive oil extraction process in an olive oil press mill. Journal of the Science of Food and Agriculture. 2021;101(3):1119-26.
39. Abdallah M, Marzocco S, Adesso S, Zarrouk M, Guerfel M. Olive oil polyphenols extracts inhibit inflammatory markers in J774A. 1 murine macrophages and scavenge free radicals. Acta Histochemica. 2018;120(1):1-10.
40. Abd Elmaksoud HA, Motawea MH, Desoky AA, Elharrif MG, Ibrahimi A. Hydroxytyrosol alleviate intestinal inflammation, oxidative stress and apoptosis resulted in ulcerative colitis. Biomedicine & Pharmacotherapy. 2021;142:112073.
41. Bulut O, Ecer Ö, Akkoç AN, Yalçın S. Healing and Antimicrobial Effects of Olea europaea L (Memecik olive oil) on Wounds in Rats. Animal Health Production and Hygiene. 2024;13(1):9-15.
42. Alkhalifa AE, Al-Ghraiybah NF, Kaddoumi A. Extra-Virgin Olive Oil in Alzheimer's Disease: A Comprehensive Review of Cellular, Animal, and Clinical Studies. International Journal of Molecular Sciences. 2024;25(3):1914.
43. Millman JF, Okamoto S, Teruya T, Uema T, Ikematsu S, Shimabukuro M, et al. Extra-virgin olive oil and the gut-brain axis: influence on gut microbiota, mucosal immunity, and cardiometabolic and cognitive health. Nutrition reviews. 2021;79(12):1362-74.

44. Vijakumaran U, Goh N-Y, Razali RA, Abdullah NAH, Yazid MD, Sulaiman N. Role of Olive Bioactive Compounds in Respiratory Diseases. Antioxidants. 2023;12(6):1140.
45. Montalbano AM, Di Sano C, Albano GD, Gjomarkaj M, Ricciardolo FLM, Profita M. IL-17A Drives Oxidative Stress and Cell Growth in A549 Lung Epithelial Cells: Potential Protective Action of Oleuropein. Nutrients. 2024;16(13):2123.
46. Yakut S, Gelen V, Kara H, Özkanlar S, Yeşildağ A. Silver Nanoparticles Loaded With Oleuropein Alleviates LPS-Induced Acute Lung Injury by Modulating the TLR4/P2X7 Receptor-Mediated Inflammation and Apoptosis in Rats. Environmental Toxicology. 2024.
47. Ben Attia T, Nahdi A, Horchani M, Elmay MV, Ksentini M, Ben Jannet H, et al. Olea europaea L. leaf extract mitigates pulmonary inflammation and tissue destruction in Wistar rats induced by concurrent exposure to noise and toluene. Drug and Chemical Toxicology. 2024:1-15.
48. Alganabi M, Lee C, Bindi E, Li B, Pierro A. Recent advances in understanding necrotizing enterocolitis. F1000Research. 2019;8.
49. Wang K, Tao G, Sylvester KG. Recent advances in prevention and therapies for clinical or experimental necrotizing enterocolitis. Digestive diseases and sciences. 2019;64:3078-85.
50. Travers CP, Carlo WA, McDonald SA, Das A, Bell EF, Ambalavanan N, et al. Mortality and pulmonary outcomes of extremely preterm infants exposed to antenatal corticosteroids. American journal of obstetrics and gynecology. 2018;218(1):130. e1-. e13.
51. Jia H, Sodhi CP, Yamaguchi Y, Lu P, Ladd MR, Werts A, et al. Toll like receptor 4 mediated lymphocyte imbalance induces nec-induced lung injury. Shock. 2019;52(2):215-23.
52. Jia S, Meng A. TGFβ family signaling and development. Development. 2021;148(5). Epub 20210312. doi: 10.1242/dev.188490. PubMed PMID: 33712443.
53. Nolte M, Margadant C. Controlling immunity and inflammation through integrin-dependent regulation of TGF-β. Trends in cell biology. 2020;30(1):49-59.
54. Lodyga M, Hinz B, editors. TGF-β1–a truly transforming growth factor in fibrosis and immunity. Seminars in cell & developmental biology; 2020: Elsevier.
55. Xin X, Cheng X, Zeng F, Xu Q, Hou L. The Role of TGF-β/SMAD Signaling in Hepatocellular Carcinoma: from Mechanism to Therapy and Prognosis. Int J Biol Sci. 2024;20(4):1436-51. Epub 20240204. doi: 10.7150/ijbs.89568. PubMed PMID: 38385079; PubMed Central PMCID: PMCPMC10878151.
56. Wagener BM, Hu M, Zheng A, Zhao X, Che P, Brandon A, et al. Neuronal Wiskott–Aldrich syndrome protein regulates TGF-β1–mediated lung vascular permeability. The FASEB Journal. 2016;30(7):2557.
57. Wesselkamper SC, Case LM, Henning LN, Borchers MT, Tichelaar JW, Mason JM, et al. Gene expression changes during the development of acute lung injury role of transforming growth factor β. American journal of respiratory and critical care medicine. 2005;172(11):1399-411.
58. Wang Ey, Chen H, Sun Bq, Wang H, Qu HQ, Liu Y, et al. Serum levels of the IgA isotype switch factor TGF-β1 are elevated in patients with COVID-19. Febs Letters. 2021;595(13):1819-24.
59. Timurkaan M, Ayyildiz H, Kalayci M. Can TGF-B1 Be an Important Prognostic Factor in Predicting Covid-19 Disease Severity? Fırat Üniversitesi Sağlık Bilimleri Tıp Dergisi. 2022;36(1):46-50.
60. Gabbia D, Sayaf K, Zanotto I, Colognesi M, Frion-Herrera Y, Carrara M, et al. Tyrosol attenuates NASH features by reprogramming the hepatic immune milieu. European Journal of Pharmacology. 2024;969:176453.
61. Ghazavi A, Ganji A, Keshavarzian N, Rabiemajd S, Mosayebi G. Cytokine profile and disease severity in patients with COVID-19. Cytokine. 2021;137:155323.

62. Zivancevic-Simonovic S, Minic R, Cupurdija V, Stanojevic-Pirkovic M, Milosevic-Djordjevic O, Jakovljevic V, et al. Transforming growth factor beta 1 (TGF-β 1) in COVID-19 patients: relation to platelets and association with the disease outcome. Molecular and Cellular Biochemistry. 2023;478(11):2461-71.

63. Karadeniz H, Avanoğlu Güler A, Özger HS, Yıldız PA, Erbaş G, Bozdayı G, et al. The prognostic value of lung injury and fibrosis markers, KL-6, TGF-β1, FGF-2 in COVID-19 patients. Biomarker Insights. 2022;17:11772719221135443.

64. Kasai H, Allen JT, Mason RM, Kamimura T, Zhang Z. TGF-β1 induces human alveolar epithelial to mesenchymal cell transition (EMT). Respiratory research. 2005;6:1-15.

65. Razali RA, Nik Ahmad Eid NAH, Jayaraman T, Amir Hassan MA, Azlan NQ, Ismail NF, et al. The potential of Olea europaea extracts to prevent TGFβ1-induced epithelial to mesenchymal transition in human nasal respiratory epithelial cells. BMC complementary and alternative medicine. 2018;18:1-10.

66. Kamasova T, Abreu AS, Paiva-Martins F, Belo L, Santos-Silva A, Valente MJ. MO1048 PHYTOTHERAPY ON RENAL HYPOXIA: THE PREVENTIVE EFFECTS OF HYDROXYTYROSOL-PRELIMINARY IN VITRO RESULTS. Nephrology Dialysis Transplantation. 2021;36(Supplement_1):gfab113. 004.

67. Toteda G, Perri A, Lupinacci S, Vizza D, La Russa A, Lofaro D, et al. FP058 AN OLIVE LEAF EXTRACT REDUCES INFLAMMATION AND FIBROSIS IN AN IN VITRO MODEL OF AUTOSOMAL DOMINANT POLYCYSTIC KIDNEY DISEASE. Nephrology Dialysis Transplantation. 2019;34(Supplement_1):gfz106. FP058.

.

Fig-1

Click here to access/download;Figure;fig-1.png

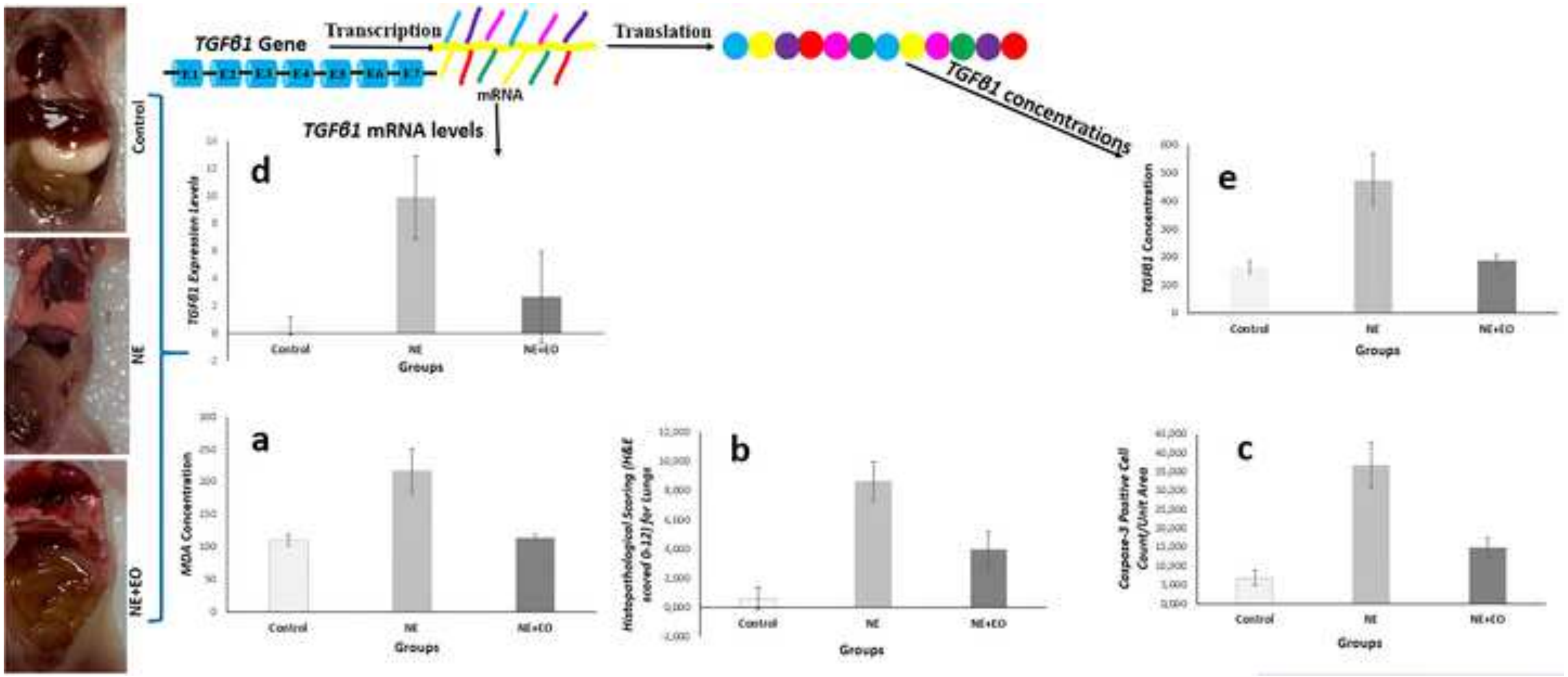

Click here to access/download;Figure;fig-2.png

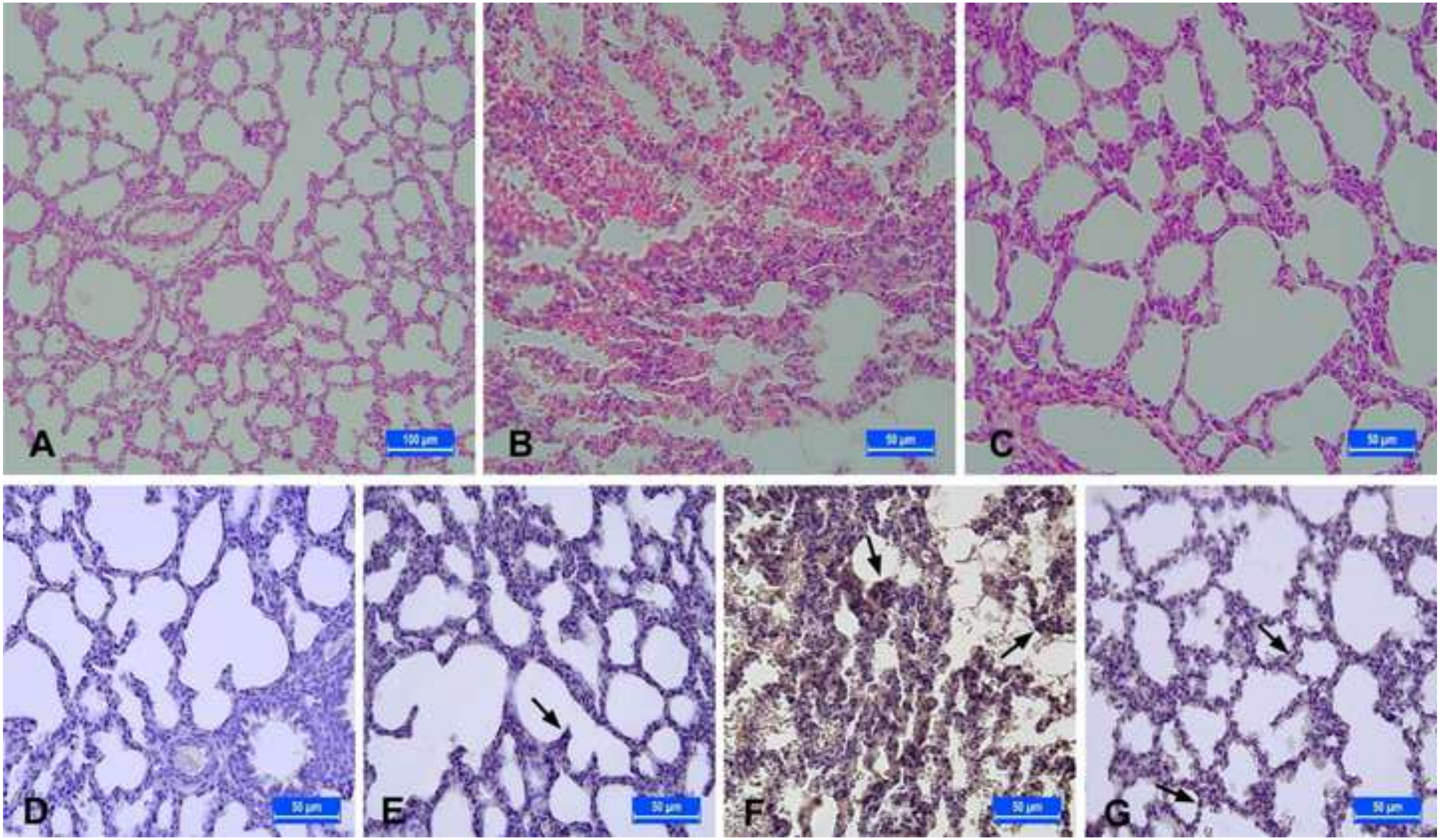

Fig-3

Click here to access/download;Figure;fig-3.png

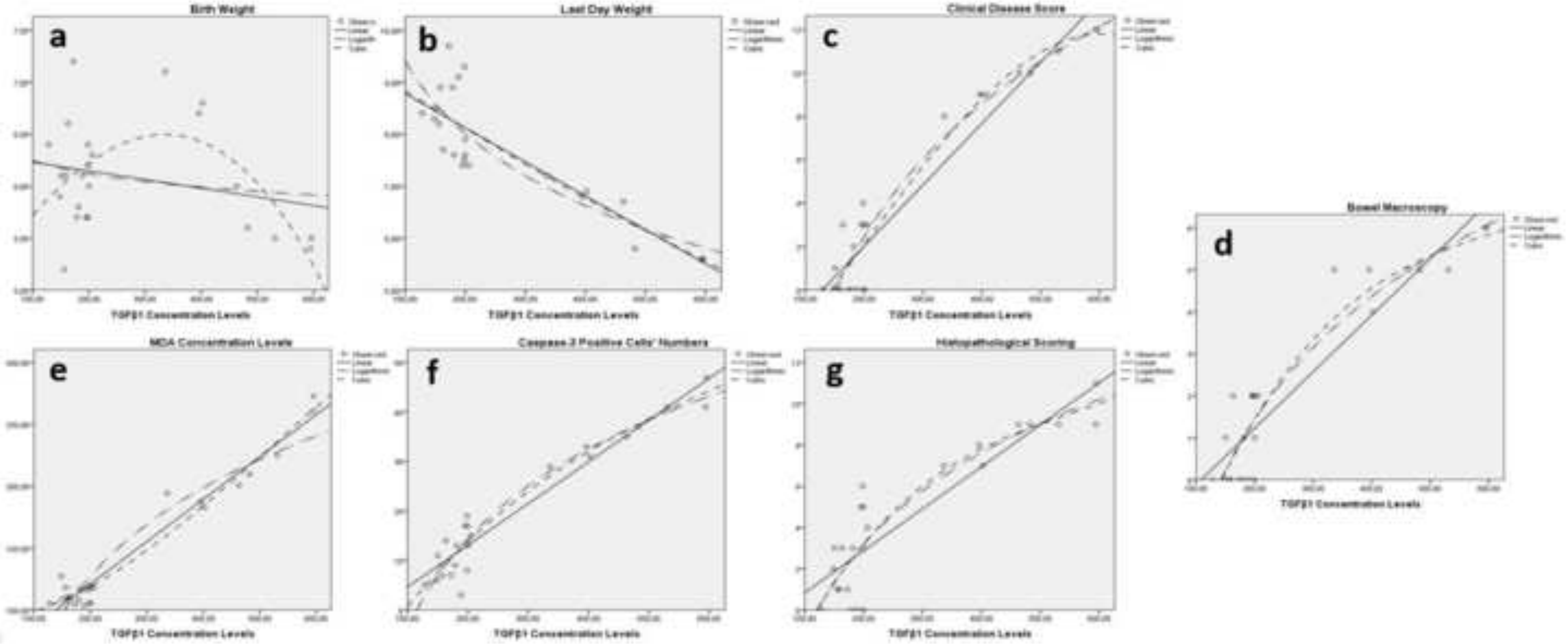

Fig-4

Click here to access/download;Figure;fig-4.png

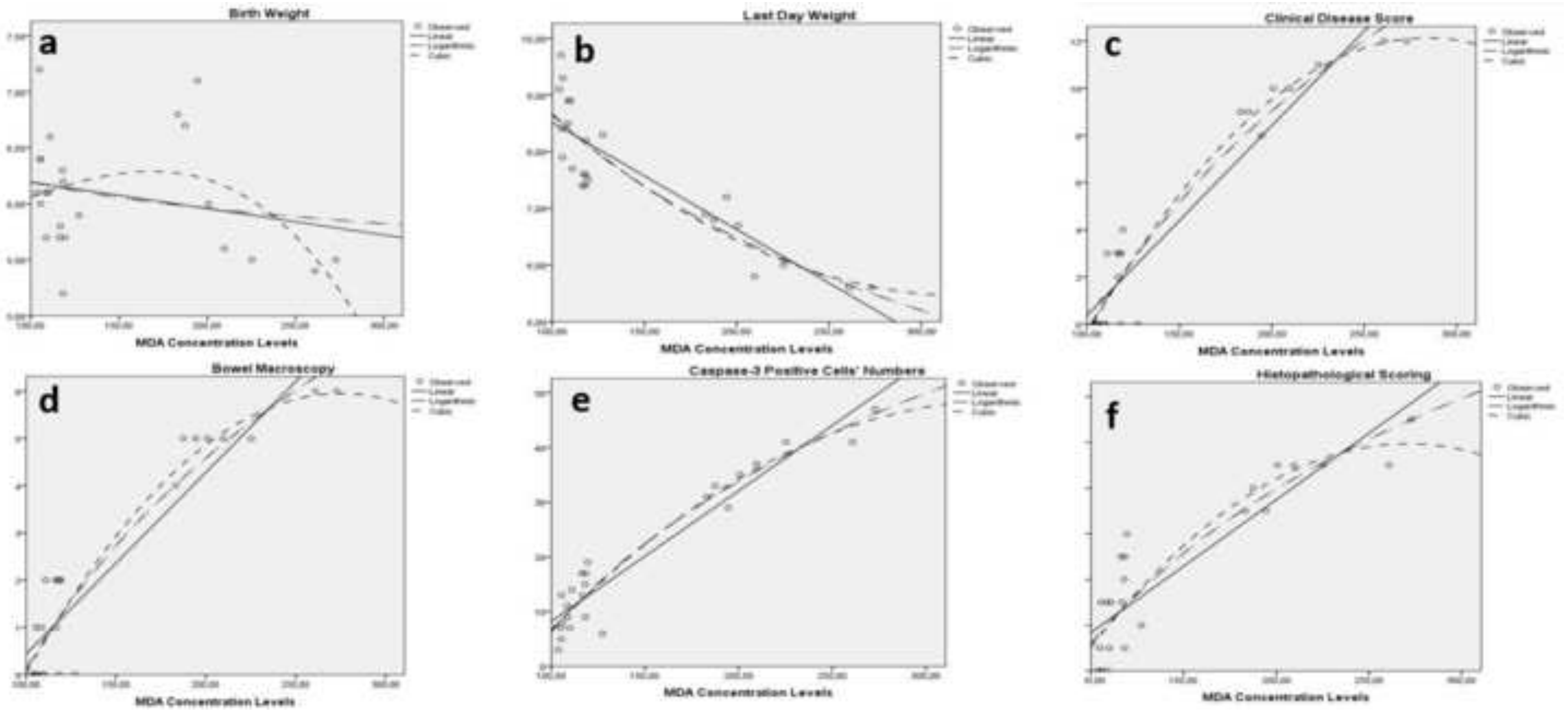

Fig-5 Click here to access/download;Figure;fig-5.png

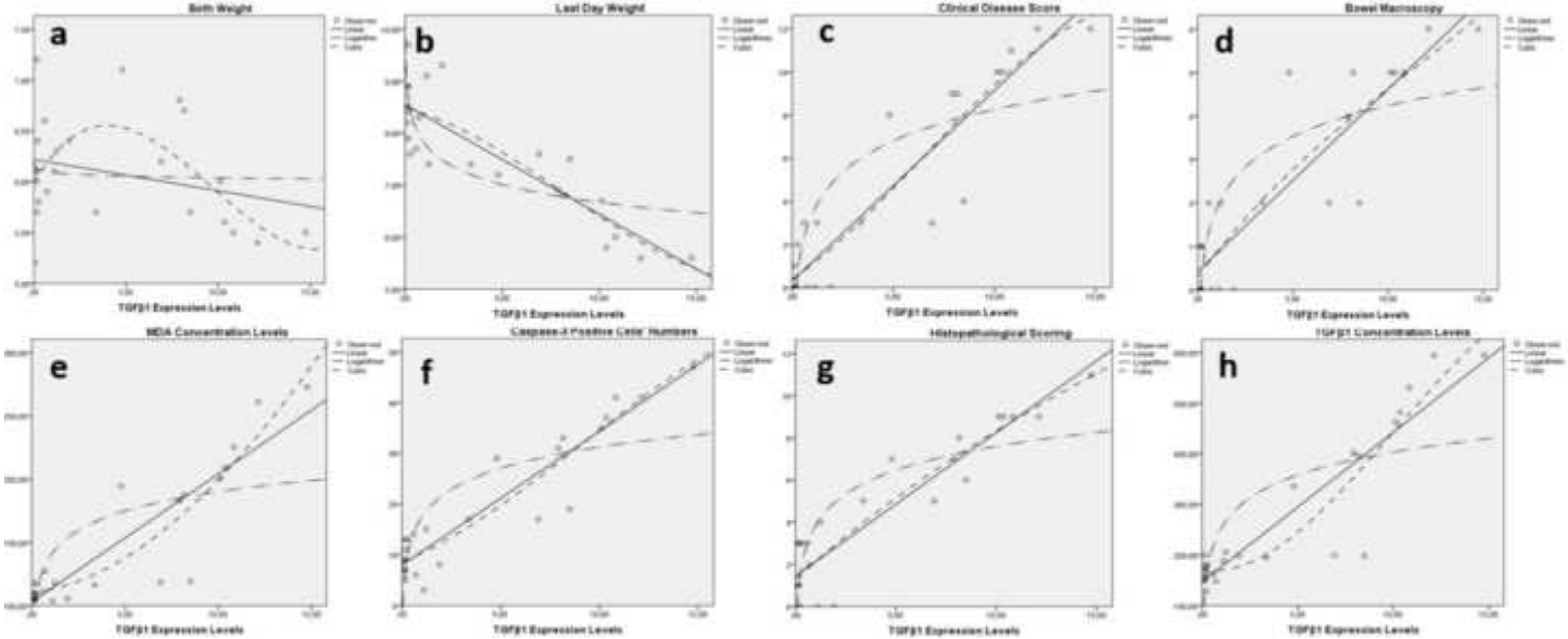

Supplement: Attachment — Submitted filename: PONE-D-24-41150_reviewer.pdf [file pone.0320938.s010.pdf]
