# Supplementary material for: Extra virgin olive oil mitigates lung injury in necrotizing enterocolitis: Effects on TGFβ1, Caspase-3, and MDA in a neonatal rat model
Source: PLoS One. 2025 Apr 15;20(4):e0320938. doi: 10.1371/journal.pone.0320938 (PMC11999148; doi:10.1371/journal.pone.0320938)
Supplement: Attachment — Submitted filename: Response_to_Reviewers_auresp_2.docx [file pone.0320938.s013.docx]

**Dear Reviewer 1**

**General Evaluation:**

**C1:** Added Clarifications: Detailed explanations for histopathological scoring methods, with Table 2 summarizing lung injury parameters, enhance reproducibility.

**R1:** These sentences were added in Histopathologic evalution of lung nad marked with yellow.

Scores from 0 (normal) to 2 (“acute lung injury”).

**C2:** Improved Methods: Revised descriptions of immunohistochemical staining protocols (e.g., antigen retrieval via citrate buffer, DAB chromogen use) provide better clarity. Although negative controls are mentioned, their representative images should clearly demonstrate the absence of nonspecific staining.

**R2:** “Arrowhead: Caspase-3 negative cells”. These sentences were added in Figure 3 and marked yellow.

Figure 3 was revised.

**Results and Discussion**

**C1:** The discussion aligns findings with the known anti-inflammatory and antioxidant properties of EO but could benefit from deeper insights into mechanisms.

**R1:** The deeper insights into anti-inflammatory and antioxidant mechanisms of EO were given into and marked with yellow in the discussion section.

**C2:** While some comparisons to existing literature are included, more detailed discussion of studies with similar interventions (e.g., other antioxidants) is warranted

**R2:** The other studies with similar interventions (e.g., other antioxidants) were given as more detailed and marked with yellow in the text.

**C3:** The conclusion should emphasize how the results could translate into clinical applications and outline future research on optimizing EO dosage and duration.

**R3:** The clinical applications and importance of the future research on optimizing EO dosage and duration were emphasized and marked yellow in the conclusion section.

**Language and Style**

**C1:** Some sentences in the discussion remain verbose and could benefit from further editing for conciseness and clarity.

**R1:** The necessery corrections were carried out according to reviewer-1 comment and marked with yellow in the text.

**Recommendations**

**C1:** The revisions significantly improve the manuscript’s scientific rigor and alignment with journal standards. However, minor additional refinements (figure quality, inter-rater reliability, and a concise discussion) are recommended to further enhance clarity and reproducibility.

**R1:** The quality of the figures was improved and checked to ensure that they met PLOS requirements by uploading them to https://pacev2.apexcovantage.com, the Preflight Analysis and Conversion Engine (PACE) digital diagnostic tool that helps ensure that figures (including figure 2) meet PLOS requirements.
