# Supplementary material for: Extra virgin olive oil mitigates lung injury in necrotizing enterocolitis: Effects on TGFβ1, Caspase-3, and MDA in a neonatal rat model
Source: PLoS One. 2025 Apr 15;20(4):e0320938. doi: 10.1371/journal.pone.0320938 (PMC11999148; doi:10.1371/journal.pone.0320938)
Supplement: Attachment — Submitted filename: Referee Report for Manuscript PONE.docx [file pone.0320938.s012.docx]

**Referee Report for Manuscript PONE-D-24-41150R1**

**Title:** *Extra virgin olive oil mitigates lung injury in necrotizing enterocolitis: effects on TGFβ1, Caspase-3, and MDA in a neonatal rat model*

**General Evaluation:**

The revised sections incorporate significant updates addressing the reviewers’ feedback. Below, I provide a detailed evaluation of the changes highlighted in yellow and their alignment with the feedback provided by the reviewers.

Added Clarifications: Detailed explanations for histopathological scoring methods, with Table 2 summarizing lung injury parameters, enhance reproducibility.

Improved Methods: Revised descriptions of immunohistochemical staining protocols (e.g., antigen retrieval via citrate buffer, DAB chromogen use) provide better clarity. Although negative controls are mentioned, their representative images should clearly demonstrate the absence of nonspecific staining.

The revised tables summarizing lung histopathological scores (Table 4) and statistical models (Table 6) align well with the manuscript’s findings.

**Results and Discussion**

The significant reduction in TGFβ1, MDA, and Caspase-3 levels in the EO-treated group provides strong evidence for the protective effects of EO.

The discussion aligns findings with the known anti-inflammatory and antioxidant properties of EO but could benefit from deeper insights into mechanisms.

While some comparisons to existing literature are included, more detailed discussion of studies with similar interventions (e.g., other antioxidants) is warranted.

The conclusion should emphasize how the results could translate into clinical applications and outline future research on optimizing EO dosage and duration.

**Language and Style**

Some sentences in the discussion remain verbose and could benefit from further editing for conciseness and clarity.

**Recommendations**

The revisions significantly improve the manuscript’s scientific rigor and alignment with journal standards. However, minor additional refinements (figure quality, inter-rater reliability, and a concise discussion) are recommended to further enhance clarity and reproducibility.
